# Neutron, X-ray diffraction, DSC, Raman, Mössbauer and leaching studies of iron phosphate glasses and crystalline phases

**DOI:** 10.1039/d5ra00295h

**Published:** 2025-02-17

**Authors:** Kajal Dadwal, Margit Fábián, Istvan Tolnai, Suruchi Sharma, Rajinder Kaur, Maria Gracheva, Krisztina Kovács, Zoltán Klencsár, Atul Khanna

**Affiliations:** a Sensors and Glass Physics Laboratory, Department of Physics, Guru Nanak Dev University Amritsar 143005 Punjab India atul.phy@gndu.ac.in; b HUN-REN Centre for Energy Research Konkoly-Thege Miklós út 29-33 1121 Budapest Hungary; c Institute of Chemistry, ELTE Eötvös Loránd University 1117-Budapest, Pázmány P. s. 1/A Hungary

## Abstract

*x*Fe_2_O_3_–(100 − *x*)P_2_O_5_ glasses were synthesized by melt quenching and structure–property correlation studies were carried. Glasses containing 25 to 40 mol% Fe_2_O_3_ were prepared while the sample with 50 mol% Fe_2_O_3_ formed a crystalline sample containing Fe_3_^2+^Fe_4_^3+^[PO_4_]_6_^3−^ and Fe_2_^2+^[P_2_O_7_]^4−^ phases on melt-quenching. Glass density increases from 2.98 to 3.20 g cm^−3^, ionic packing fraction is in the range of 0.63–0.65 and the glass transition temperature decreases from 500 °C to 493 °C on increasing Fe_2_O_3_ concentration from 25 to 40 mol%. Pair distribution function analysis and Reverse Monte Carlo simulations of neutron diffraction datasets were used to calculate the atomic pair distributions, interatomic distances and co-ordination environments. The P–O co-ordination is essentially tetrahedral and is in the range: 3.9–3.7 (±0.1), the Fe–O co-ordination number decreases steadily from 4.8 to 4.2 (±0.1) with an increase in Fe_2_O_3_ concentration in the phosphate network, while O–O co-ordination is in the range: 6.6–6.3(±0.1), the decrease in these co-ordination numbers are due to an increase in oxygen deficiency in the glass network with an increase in Fe_2_O_3_ mol%. Fe–O and P–O pair distributions are asymmetrical indicating short-range disorder due to the existence of a wide range of bond-lengths with maxima at 1.79 Å and in the range: 1.45–1.51 Å respectively. Mössbauer studies carried out at room temperature and 80 K found that Fe exists in 2+ and 3+ valence states, and the glass and crystalline samples contained Fe^2+^ at least at three different sites. Raman studies found that the *meta* and pyrophosphate structural units are dominant species up to 35 mol% Fe_2_O_3_ concentration, while the orthophosphate units are in majority at 40 mol% of Fe_2_O_3_. The crystalline sample is a two phase material and contained both orthophosphate and pyrophosphate units with the former being the dominant species. Leaching studies on two iron phosphate glasses carried out in purified water at 90 °C found that dissolution of glasses decreases and the chemical durability increases drastically with an increase in Fe_2_O_3_ mol%.

## Abstract 2

*x*Fe_2_O_3_–(100 − *x*)P_2_O_5_ glasses were synthesized by melt quenching and structure–property correlation studies were carried.

## Graphical abstract

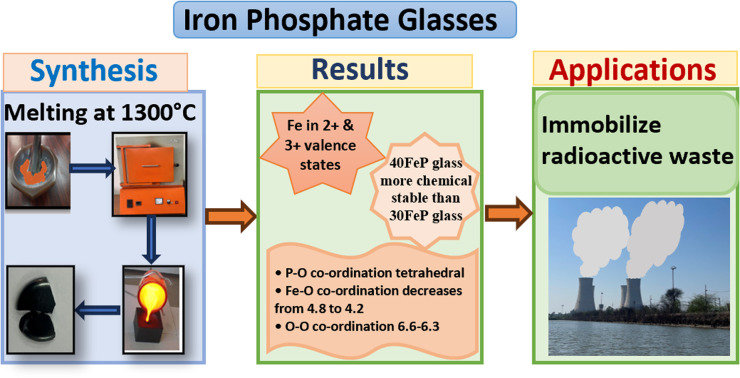

## 1. Introduction

Nuclear waste management is an end stage of the nuclear fuel cycle and it is important to find matrices or materials that can be used to incorporate and immobilize high level radioactive waste.^1,2^ Borosilicate glasses have been widely used for high-level nuclear waste immobilization.^3–6^ However, the high-level waste containing halide and Mo ions, P and noble metal atoms are only sparingly soluble in borosilicate glasses, and this limits the waste loading, and increases the cost and time of vitrification.^7^ Iron phosphate and lead iron phosphate glasses are functional materials that are considered to be economical alternatives to borosilicate glasses for nuclear waste immobilization,^8–12^ because these are good solvents for heavy metal ions and possess excellent leaching resistance in acidic and neutral medium.^13^ Moreover, the addition of MoO_3_ enhances the thermal stability and water durability of iron phosphate glasses.^14^ The iron phosphate glasses are chemically more durable than alkali and alkaline-earth phosphate glasses due to the existence of Fe–O–P linkages, the Fe^2+^/Fe^3+^ in the phosphate glass network strengthens the cross-linking between the polyphosphate chains and enhances the chemical and thermal durability of glasses.^15,16^ The open structure and voids in iron phosphate glass network promotes the incorporation of large concentration of actinides and nuclear waste.^17^

The basic building blocks of the glassy and crystalline phosphates are the P–O tetrahedral structural units that are connected by covalently bonded oxygens and that form various phosphate anions. The tetrahedra are categorized using the *Q*^*i*^ terminology where *i* represents the number of bridging oxygens per tetrahedron. The three phosphate polyhedral units are: *Q*^0^, *Q*^1^ and *Q*^2^ respectively.^18^ The number of tetrahedral linkages through bridging oxygens between neighbouring P-tetrahedra can be classified by the oxygen to-phosphorus (O/P) ratio. Glass systems with O/P = 4, 3.5 and 3 are represented as *Q*^0^, *Q*^1^ and *Q*^2^ polyhedra and termed as orthophosphate, pyrophosphate and metaphosphate units respectively. The metaphosphates possess long chains of P-tetrahedra linked through two bridging oxygens, the pyrophosphates with single bridging oxygen terminates these chains and forms (P_2_O_7_)^4−^ anions while the orthophosphates without any bridging oxygens are similar to isolated phosphate groups that exist in the crystalline phosphate compounds.^18–20^

The chemical durability of phosphate glasses can be enhanced considerably by adding Al_2_O_3_ and Fe_2_O_3_ in the glass network; the addition of these metal oxides replaces the easily hydrated –P–O–P– bonds by more hydrate resistant –M–O–P– bonds, where M are the metal cations.^15,16^ The glass formation range of *x*Fe_2_O_3_–(100 − *x*)P_2_O_5_ system is reported to be 10 to 45 mol% of Fe_2_O_3_.^21^ The 40Fe_2_O_3_–60P_2_O_5_ glass is reported to exhibit the best chemical durability or lowest leaching rate among all the iron phosphate glasses,^22–26^ leaching is an important property of glasses/matrices that needs to be evaluated for their application in nuclear waste immobilization.

As the concentration of Fe_2_O_3_ increases in the phosphate network, the number of Fe–O–P linkages increases at the expense of P–O–P bonds and, the chain-like metaphosphate network breaks into dimeric pyrophosphate and finally into isolated orthophosphate groups.^19,20^ Iron phosphate glasses are reported to contain both Fe^2+^ and Fe^3+^ in the network, and the valence state of Fe is reported to depend on the use of either FeO or Fe_2_O_3_ as starting material in the synthesis of iron phosphate glasses.^21^ Molecular dynamics (MD) studies using Buckingham potential were carried out on both *x*FeO–(100 − *x*)P_2_O_5_ and *x*Fe_2_O_3_–(100 − *x*)P_2_O_5_ glass systems, and Fe–O, P–O and O–O atomic pair correlations were determined.^27^ The experimental studies in which iron phosphate glasses were synthesized by melt-quenching have also reported large effects of melting temperature and melting time on relative concentration of Fe^2+^ and Fe^3+^ in the structure,^19^ however several of these experimental results can be erroneous as the investigators employed alumina or silica crucibles instead of more durable platinum crucibles for the synthesis of glasses.^19,21,28,29^ It is well established that alumina and silica crucibles can introduce significant concentration of Al^3+^ and Si^4+^ impurities in the oxide glass melt and give rise to the impurity Al–O and Si–O peaks in the total pair correlation functions and consequently give incorrect results on Fe–O and P–O co-ordination numbers and bond lengths since the Fe–O and P–O peaks exist at almost the same positions as Al–O and Si–O peaks respectively.

It is therefore important that iron phosphate glasses are prepared by melting the batch materials in more durable platinum crucible for the accurate determination of glass structure and properties. In the present study series of iron phosphate glasses were prepared by melting the starting materials in platinum crucibles and subsequently characterized by density measurements, X-ray diffraction, differential scanning calorimetry (DSC), Raman and Mössbauer spectroscopy, neutron diffraction, pair distribution function and Reverse Monte Carlo (RMC) simulations and leaching tests. The RMC analysis was used to calculate the partial atomic pair distributions, the cation–oxygen bond lengths and Fe–O and P–O co-ordination numbers; RMC simulations give accurate and reliable values of glass short-range structural properties since it does not suffer from the disadvantages of overlapping atomic pair correlation peaks in the total pair distribution function,^30–34^ that are difficult to deconvolute unambiguously when different atomic pairs exist at same or very similar distances. The chemical durability of two iron phosphate glasses were studied by measuring their leaching characteristics in MQ water at 90 °C for a period of 10 days.

## 2. Experimental

### 2.1 Glass synthesis

Disk shaped samples of the system: *x*Fe_2_O_3_–(100 − *x*)P_2_O_5_ (*x* = 25, 30, 35, 40 and 50 mol%) were prepared by the melt-quenching (hereafter referred as 25FeP, 30FeP, 35FeP, 40FeP and 50FeP respectively). The starting materials for glass preparation were Fe_2_O_3_ (Aldrich India, 99%) and NH_6_PO_4_ (99%, Otto Kemi, India). The net weight of the batch mixture for all the samples was about 10 g. Appropriate amounts of materials were weighed and ground in an agate mortar-pestle for about 30 min. The batch mixture was then transferred into a platinum crucible. It was first heated in an electric furnace at 200 °C for 1 h and subsequently the temperature of the furnace was slowly increased to 550 °C to remove the residual gases (NH_3_ and H_2_O). The material was kept at this temperature for ∼1 h. Afterwards, the mixture was melted in another furnace and heated to 1300 °C, the melt was kept at this temperature for 1 h. The melt was then poured onto a brass block to get the final disk-shaped glass samples. All the glass samples were shining black in color. Samples upto 40 mol% Fe_2_O_3_ formed glasses, however the sample with 50 mol% Fe_2_O_3_ in the starting batch mixture did not produce a glass but solidified into a crystalline material on melt-quenching as confirmed by X-ray and neutron diffraction studies discussed below.

### 2.2 Density measurements

The density, *ρ* of the disk-shaped glasses was measured by the Archimedes method using dibutylphatalate (DBP) as the immersion fluid on an electronic balance of sensitivity 10^−4^ g at laboratory temperature by using the following relation:1
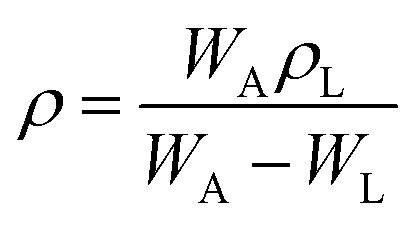
where *ρ*_L_ is the density of the DBP oil at the laboratory temperature, *w*_A_ and *w*_L_ are the weight of the glass sample in air and the liquid, respectively. The maximum error in density was in the range of ± 0.01 to 0.07 g cm^−3^. The density values were used to calculate molar volume, 
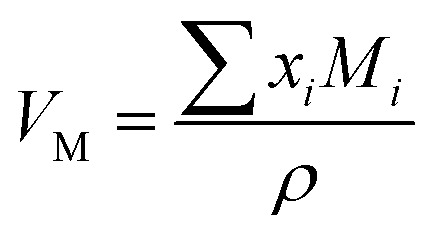
 (where *x*_*i*_ is the mol% of *i*th component and *M*_*i*_ is its molecular weight). The packing fraction, PF, of glasses is the ratio of ionic volume to molar volume and calculated by using the following formula:^35^2
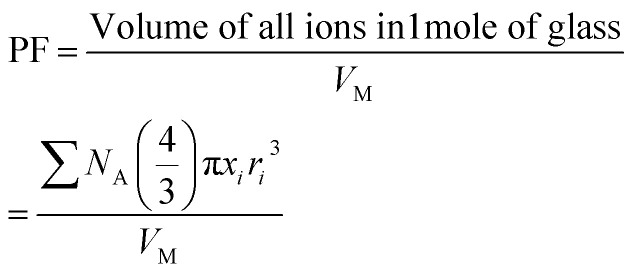
where *x*_*i*_ and *r*_*i*_ are mole fraction and the ionic radius of the *i*th ion in one formula unit of glass sample. The values of ionic radii were taken as: *r*_Fe_^3+^ = 0.55 Å, *r*_O_^2−^ = 1.40 Å and *r*_P_^5+^ = 0.38 Å.^36^

### 2.3 X-ray diffraction experiments

X-ray diffraction (XRD) studies were carried out on powdered samples on Bruker D8 Focus XRD system with Cu K_α1,2_ radiation in the *2θ* range of 10–90°. The X-ray tube was operated at 40 kV and 30 mA and the scattered X-ray intensity was measured with a scintillation detector using step size of 0.02° and scan rate of 0.5° per min.

### 2.4 Differential scanning calorimetry

The thermal parameters such as glass transition temperature, *T*_g_, crystallization temperature, *T*_c_ and the melting temperature, *T*_m_ were determined on SETARAM Labsys Evo DSC system. About 50 mg powder sample was taken in a platinum pan and the differential heat flow scan was recorded at a heating rate of 10 °C min^−1^ in the temperature range of 200–1200 °C under argon flow conditions (10 ml min^−1^). The glass transition temperature was taken as the mid-point value. The peak values of exothermic crystallization and endothermic melting peaks were taken as the crystallization and melting temperatures respectively. The maximum uncertainty in the measurement of *T*_g_, *T*_c_ and *T*_m_ is ± 1 °C.

### 2.5 Raman spectroscopy

Raman scattering studies were performed at room temperature on the iron phosphate samples on Renishaw In-Via Reflex micro-Raman spectrometer in the Raman shift range of 30–1500 cm^−1^ at a spectral resolution of 0.5 cm^−1^. The Stokes spectra were recorded by using a 514.5 nm argon-ion excitation laser, an edge filter, diffraction grating with 2400 lines per mm and a CCD detector.

### 2.6 Neutron diffraction experiments

The neutron diffraction studies were carried out on powdered iron phosphate samples at the 2-axis Position Sensitive Detector (PSD) diffractometer, of Budapest Neutron Centre (BNC), Hungary, using monochromatic thermal neutrons of de Broglie wavelength, *λ* = 1.069 Å in the scattering vector or momentum transfer function (*Q*) range of 0.45 to 9.8 Å^−1^. The pulverized samples of mass 4 to 5 g were placed in thin-walled cylindrical vanadium can of diameter of 8 mm and the diffraction data was recorded for 24 h each. The raw neutron diffraction data of four glass samples was corrected for background, absorption and multiple scattering and normalized with vanadium rod standard. The structure factors, *S*(*Q*)'s were calculated from the raw diffraction data using the programme package available at the BNC facility.

### 2.7 Pair distribution function (PDF) by Fourier transformation

The atomic pair distribution function, *g*(*r*) of each sample was calculated by the Fourier sine transformation of the neutron interference function amplified with *Q i.e. F*(*Q*)*= Q*(*S*(*Q*) − 1) by using the following formula:^30–34^3
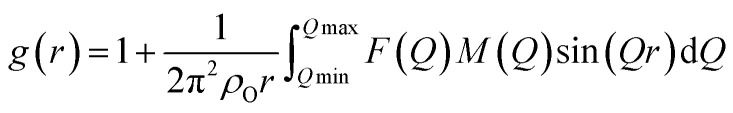
where, *ρ*_o_ is the atomic number density of the sample and *M*(*Q*) is the Lorch modification function defined as below:
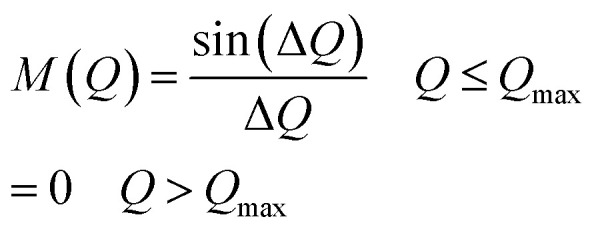
and
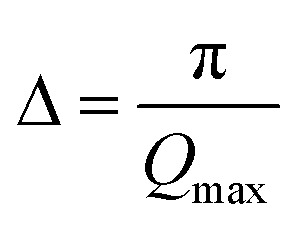
*Q*_max_ was selected around 9.8 Å^−1^ for calculating *g*(*r*) by (3).

### 2.8 Reverse Monte Carlo (RMC) simulations

The experimental neutron structure factors, *S*(*Q*) were simulated by the RMC technique using the RMC++ Version 1.5.1 software package.^37–40^ During the RMC simulation runs, the partial structure factors, *S*_*ij*_(*Q*) were calculated from the pair distribution functions *g*_*ij*_(*r*) by Fourier transformation. If *k* is the total number of elements in the sample (*k* = 3 in the present glass system), then *k*(*k* + 1)/2 = 6 different atom pairs exist in its glass structure. The RMC technique minimizes the squared difference between the experimental *S*(*Q*) and the simulated one from a 3-dimensional atomic configuration by using the following equations4
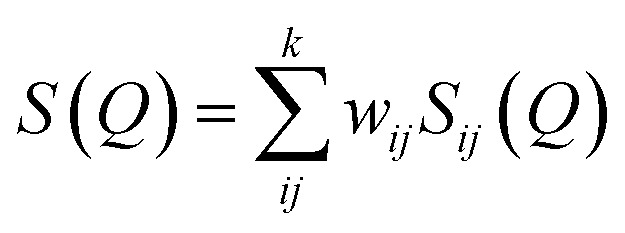
5
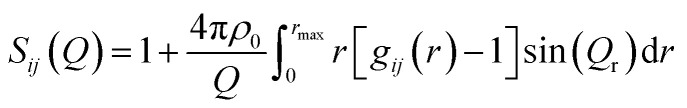
6
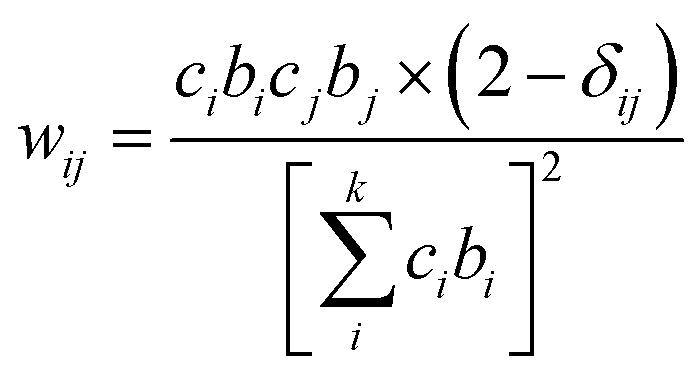
where *c*_*i*_, *b*_*i*_ are the molar fraction and coherent neutron scattering length for atoms of type *i*, the *S*_*ij*_(*Q*) is the partial structure factors and *w*_ij_ are the neutron scattering weight factors for the 6 atomic pairs: Fe–Fe, Fe–P, Fe–O, P–P, P–O and O–O of the glass system. *g*_*ij*_(*r*) are the partial pair correlation functions for the above mentioned 6 atomic pairs and these give the measure of the probability of finding the *j*th atom at a distance, *r* from the *i*th atom.

The RMC simulations were started by building an initial random atomic configuration in a simulation box that was populated with 10 000 atoms of Fe, P and O. The atomic number densities, *ρ*_o_ were 0.0799, 0.0818, 0.0813 and 0.0802 atoms per Å^−3^ (corresponding to box edges of 26.79, 24.81, 24.86 and 24.97 Å) for the glass samples 25FeP, 30FeP, 35FeP and 40FeP respectively. The atoms inside the box were constrained to move apart by certain minimum interatomic distances (cut-off distances) that were provided in the RMC input program. No other constraints (such as Fe–O or P–O co-ordination constraints) were imposed on the atomic pairs during the RMC simulations. Repeated RMC runs were performed by varying the cut-off distances slightly, to produce reliable data with good stability and statistics. About 20 atomic configurations were obtained from the RMC calculations of each sample corresponding to more than 1 100 000 accepted atomic displacements inside the simulation box. After multiple runs, the experimental and the RMC simulated structure factor, *S*(*Q*) matched well and the atomic pair-correlation functions, *g*_*ij*_(*r*), coordination number distributions, *N*_*ij,*_ and bond angle distribution were calculated from the RMC simulated data.

### 2.9 Mössbauer spectroscopy

^57^Fe Mössbauer spectra were recorded at room temperature (298–300 K) in transmission geometry by using a standard WissEl Mössbauer spectrometer setup along with a ^57^Co(Rh) radioactive source with an activity of ∼2.2 mCi. The source movement followed a sinusoidal velocity signal. Unfolded spectra were recorded in 2048 channels and were subsequently folded into 1024 channels for analysis. Isomer shift (*δ*) values are quoted with respect to that of α-Fe at room temperature. Circular absorbers were prepared with a diameter of 15.5 mm by using sample amounts that were expected to result in comparable (∼12 mg cm^−2^) Fe surface densities of the resulting samples based on the nominal sample stoichiometries. The powdered samples were in addition mixed in each case with 200 mg of cellulose in order to achieve a uniform absorber surface density. For the low temperature (*T* ≈ 80 K) measurements the samples were kept in a bath type cryostat (SVT-400-MOSS, Janis, Woburn, MA, USA) filled with liquid nitrogen, while the spectrometer operated with a triangular velocity waveform, in constant acceleration mode, recording spectra in 512 channels before folding. Unless explicitly noted otherwise, the resulting spectra were fitted to symmetrical quadrupole doublets by using version 4.0i of the MossWinn program.^41^

### 2.10 Leaching tests

The chemical durability of the two disk shaped bulk glass samples: 30FeP (weight = 1.772 g) and 40FeP (1.919 g) and the chemical evolution of the leaching solution in water purified with Millipore Milli-Q lab water system (Merck, USA). This purified water is referred to as MQ-water in the manuscript.

MQ-water at 90 ± 1 °C were analyzed by calculating the normalized release and glass dissolution rate of P and Fe using the equations specified in the ASTM C1285-21 protocol.^42^ The procedure used for the leaching analysis is described elsewhere^43^ and leaching tests were carried over a total period of 10 days. After 1, 2, 3, 7, and 10 days, the containers were opened, and the leachates were filtered through a 0.45 μm syringe filter. Subsequently, the leachates were acidified with cc. HNO_3_ for inductively coupled plasma optical emission spectroscopy (ICP-OES) measurements to measure the concentration of P and Fe concentration (in the units of mg l^−1^) that leached out in MQ-water using a PerkinElmer Avio 200 ICP-OES apparatus.^43^

## 3. Results and discussion

### 3.1 X-ray diffraction (XRD)

**Fig. 1:**
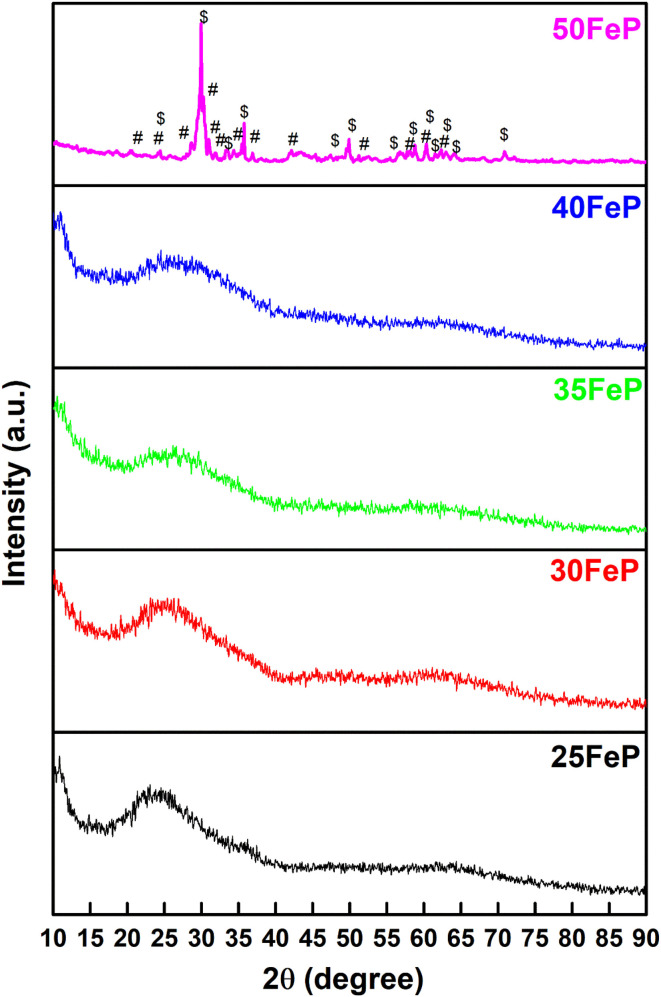
XRD patterns of iron phosphate samples. The sharp peaks in the sample 50FeP are due to the coexisting Fe_7_(PO_4_)_6_ (#) and Fe_2_P_2_O_7_ ($) crystalline phases.

The XRD patterns of all iron phosphate samples are shown in Fig. 1 and it is found that samples containing 25, 30, 35 and 40 mol% of Fe_2_O_3_ show only broad humps in the *2θ* range of 15° to 35°, which confirmed their amorphous nature.

The sample: 50FeP prepared using 50 mol% Fe_2_O_3_ in the batch mixture, shows several sharp peaks; this sample is crystalline and its XRD peaks match with those of Fe(ii)_3_Fe(iii)_4_[PO_4_]_6_ and Fe(ii)_2_[P_2_O_7_] phases both of which possess triclinic crystal structure (powder diffraction files # 72-2446 and 76-1762 respectively).

### 3.2 Density, molar volume and packing fraction

The density data of the iron phosphate samples prepared with 25, 30, 35, 40 and 50 mol% Fe_2_O_3_ is given in Table 1. Density increases from 2.98 g cm^−3^ to 3.59 ± 0.05 g cm^−3^ as the concentration of Fe_2_O_3_ is increased from 25 to 50 mol%, it may be noted that sample prepared with 50 mol% Fe_2_O_3_ in the starting batch mixture, is not a glass but is crystalline.

**Table 1:** Composition, mass density, molar volume, packing fraction and atomic number densities of iron phosphate glasses (25FeP–40FeP) and crystalline sample (50FeP)

Sample code	Composition [mol%]		Density, *ρ* [g cm^−3^]	Molar mass (amu)	Molar volume *V*_M_ [cm^3^ mol^−1^]	Packing fraction (PF)	Atomic number density *ρ*_o_ [Å^−3^]
	Fe_2_O_3_	P_2_O_5_					
25FeP	25	75	2.98	146.38	49.1	0.64	0.0799
30FeP	30	70	3.13	147.26	47.0	0.65	0.0818
35FeP	35	65	3.18	148.15	46.6	0.64	0.0813
40FeP	40	60	3.20	149.04	46.5	0.63	0.0802
50FeP	50	50	3.59	150.82	42.0	0.67	0.0861

The molar masses of Fe_2_O_3_ and P_2_O_5_ are 159.6 u and 141.9 u respectively; therefore, the replacement of lighter P_2_O_5_ by the heavier Fe_2_O_3_ molecules in the glass network produces a steady enhancement in the density. Al-Hasni *et al.*^44^ reported density of 3.03 g cm^−3^ for the 40Fe_2_O_3_–60P_2_O_5_ glass which is lower than the density of 3.20 ± 0.05 g cm^−3^ of the glass of equal composition prepared and characterized in the present study.

The molar volume, *V*_M_ defined as the volume occupied by 1 mole of glass molecules decreases from a value of 49.1 to 42.0 cm^3^, on increasing Fe_2_O_3_ concentration from 25 to 50 mol%. *V*_M_ of iron phosphate glasses is found to be significantly higher than that of other oxide glasses such as borate and borosilicate glasses, for instance lead borate,^45^ lead borosilicate,^45^ lead aluminoborosilicate,^46^ bismuth borate^47^ and bismuth borosilicate^48^ have molar volume in the range of 24.2 to 42.7 cm^3^, the alkali and alkaline-earth silicate and aluminosilicate glasses have lower *V*_M_ in the range of 16 to 30 cm^3^.^49^

The PF in iron phosphate glasses is in the range of 0.63–0.65 and it shows only a small variation with an increase in Fe_2_O_3_ concentration from 25 to 40 mol%, both *V*_M_ and PF are important structural parameters of glasses that are used as matrices for radioactive waste immobilization. A larger *V*_M_ and a smaller value of PF indicates greater concentration of voids that can incorporate other ions in the glass structure.

### 3.3 Thermal properties

**Fig. 2:**
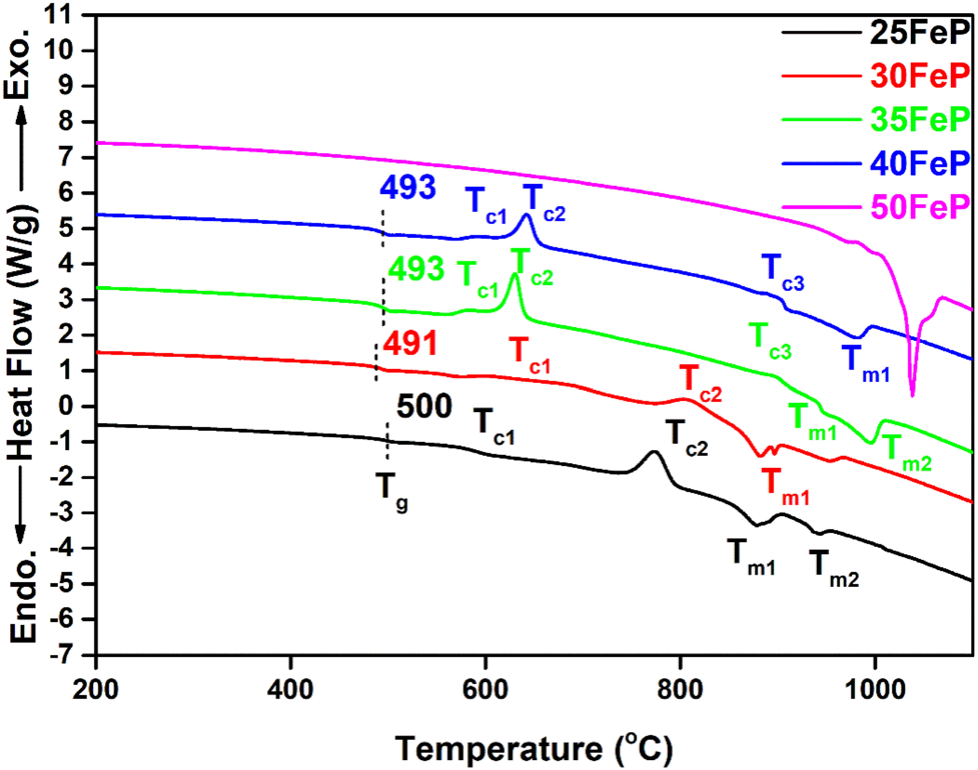
DSC curves of iron phosphate samples. The dotted line indicates the midpoint of glass transition. The sample 50FeP is crystalline and exhibits only endothermic liquidus peak.

DSC thermographs of the 4 iron phosphate glasses and one crystalline sample are displayed on Fig. 2. The glass transition temperature, *T*_g_ decreases steadily from 500 °C to 493 °C as Fe_2_O_3_ concentration is increased from 25 to 40 mol% (Table 2). This is because the weaker Fe–O (*E*_Fe–O_ = 409 kJ mol^−1^) bonds replace the significantly stronger P–O (*E*_P–O_ = 597 kJ mol^−1^) bonds in the phosphate network,^36^ and this lowers the glass transition temperature. The value of *T*_g_ (493 ± 1 °C) in 40Fe_2_O_3_–60P_2_O_5_ glass is in good agreement with the value of *T*_g_ of 498 ± 5 °C reported for the same glass by Al-Hasni *et al.*^44^

**Table 2:** Thermal properties of iron phosphate glasses

Sample	*T*_g_ [^o^C]	*T*_c_ [^o^C]			*T*_m_ [^o^C]		*E*_B_ [kJ mol^−1^]
		*T*_c1_	*T*_c2_	*T*_c3_	*T*_m1_	*T*_m2_	
25FeP	500	588	775	—	875	944	550
30FeP	491	617	812	—	884	943	540
35FeP	493	594	630	897	887	942	531
40FeP	493	587	643	899	948	990	521

The average single bond enthalpy, *E*_B_ in four glasses was calculated by using the following formula:^50^7
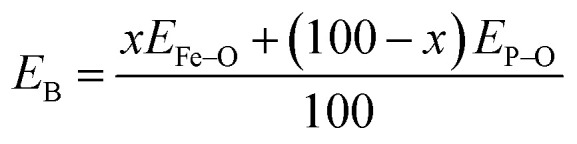
where *x* = 25, 30, 35 and 40 mol%. It is found that the *E*_B_ decreases (Table 2) with an increase in Fe_2_O_3_ content and this decreases *T*_g_ from 500 to 491 °C on increasing Fe_2_O_3_ concentration from 25 to 30 mol%, however with further increase in Fe_2_O_3_ content to 35 and 40 mol%, *T*_g_ again increases slightly to 493 °C. This indicates cross linking of phosphate chains by Fe^3+^ ions through non bridging oxygens of P–O tetrahedra at high Fe_2_O_3_ content which strengthens the glass network. The intensity of exothermic crystallization peaks increases which indicates that the crystallization tendency increases or the glass forming ability decreases with an increase in Fe_2_O_3_ concentration from 25 to 50 mol%. The DSC results are consistent with X-ray and neutron diffraction findings; the latter studies confirm that the sample with 50 mol% Fe_2_O_3_ is not a glass but is crystalline containing coexisting Fe_7_(PO_4_)_6_ and Fe_2_P_2_O_7_ phases, therefore its DSC scan did not exhibit glass transition or crystallization peaks but an endothermic melting peak at 1041 °C. Table 2 gives the thermal properties data of all the glass samples.

### 3.4 Vibrational spectra

**Fig. 3:**
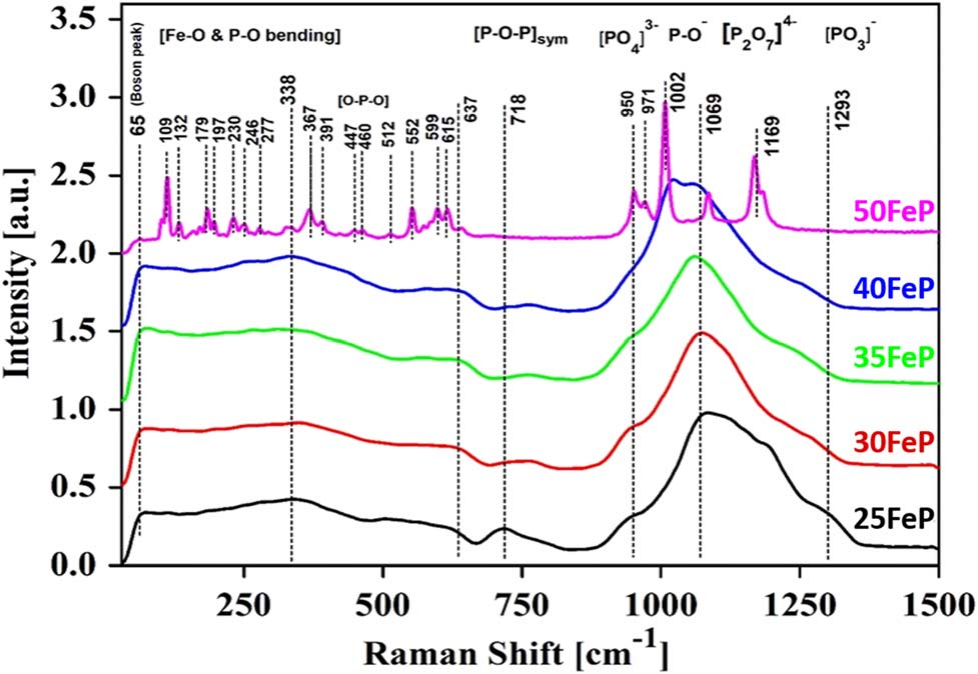
Raman spectra of four iron phosphate glasses and one crystalline sample.

**Fig. 4:**
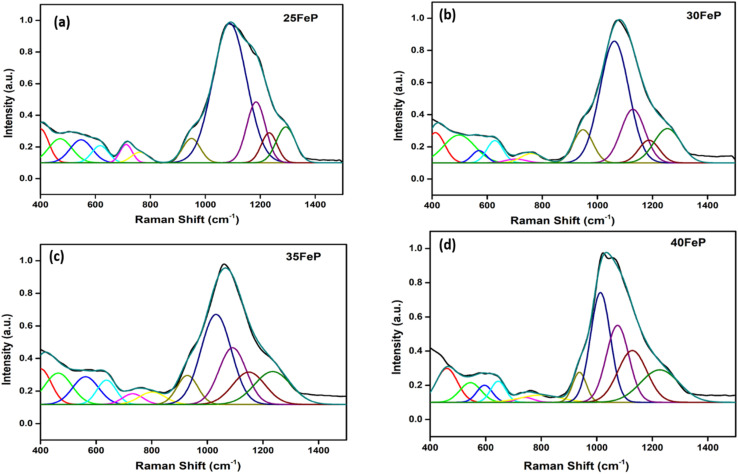
Decomposition of Raman spectrum for (a) 25FeP (b) 30FeP (c) 35FeP (d) 40FeP into Gaussian peaks.

The Raman spectra of iron phosphate samples are shown in Fig. 3. The sharp peak at low frequency of ∼65 cm^−1^ is the boson peak which is a universal feature of the disordered materials and glasses.^51–55^ This peak is not prominent in the 50FeP sample as it is crystalline and not glassy. Fig. 4 shows the Raman spectra of glassy samples; 25FeP, 30FeP, 35FeP and 40FeP deconvoluted using 11 independent Gaussian peaks in range 400–1500 cm^−1^ using peak positions at approximately same positions as reported in the earlier study by Stoch *et al.*^56^

All the peaks in the mid-frequency region below 400 cm^−1^ are due to the bending vibration modes of Fe–O and P–O bonds.^19,20^ In this frequency range, glassy samples show a broad band centred at ∼340 cm^−1^ while the Raman spectra of 50FeP glass-ceramic sample shows several sharp peaks at 109, 132, 179, 197, 230, 246, 277, 336, 367 and 391 cm^−1^.^20^ In 50FeP glass ceramic sample, peaks were observed at 447 and 460 cm^−1^ which are due to O–P–O bending modes of *Q*^0^ units.^19,20^ The Raman band in glasses in the range of 664–823 cm^−1^ is due to the symmetric stretching vibrations of P–O–P bonds that contain bridging oxygens. Relative intensity of this band decreases with decrease in concentration of P_2_O_5_. The broad band in the Raman shift range: 869–1376 cm^−1^ in glasses is due to symmetric and asymmetric stretching vibration modes of bridging and non-bridging oxygens associated with different phosphate tetrahedra *i.e.*, orthophosphate (*Q*^0^), pyrophosphate (*Q*^1^) and metaphosphate (*Q*^2^), respectively. The shoulder at ∼950 cm^−1^ is due to the symmetric stretching vibration of (PO_4_)^3−^ anions or the orthophosphate groups. In case of 50FeP sample, two sharp peaks are observed at 950 and 971 cm^−1^ and these are due to vibrations of orthophosphate, (PO_4_)^3−^ anions. The band at 1160 cm^−1^ is due to the asymmetric stretching vibrations of pyrophosphate, (P_2_O_7_)^4−^ units of the *Q*^1^ groups.^19,20^

**Fig. 5:**
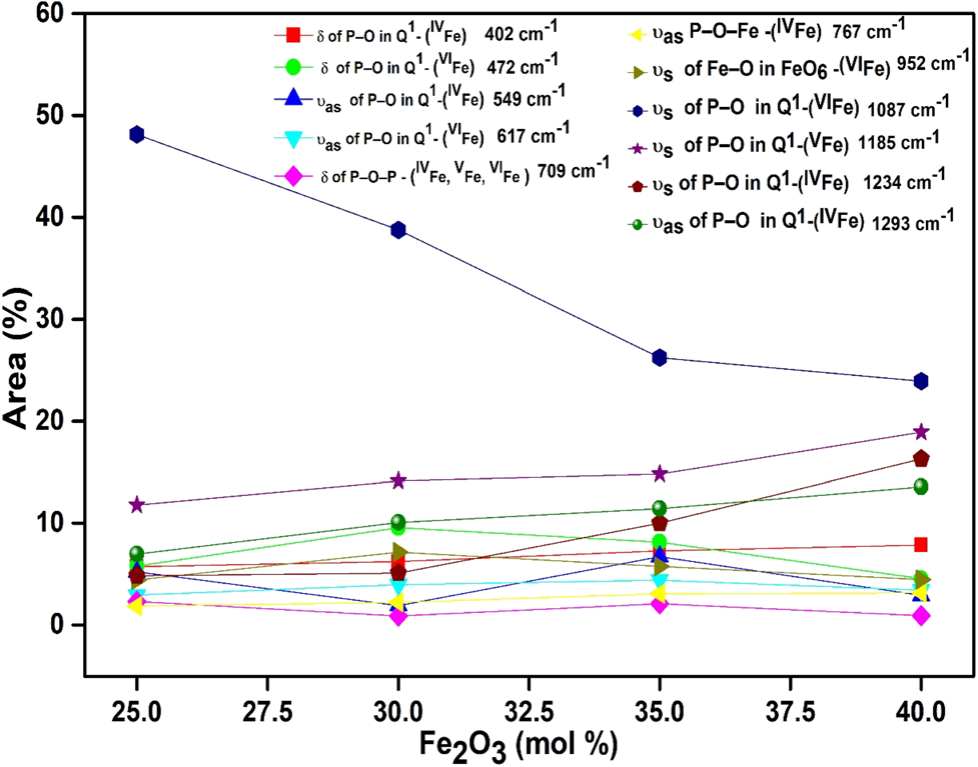
Area (%) *vs.* concentration of Fe_2_O_3_ (mol%).

In the glass samples: 25FeP, 30FeP and 35FeP, the maximum intensity is of the Raman band at 1169 cm^−1^ and this indicates that pyrophosphate groups are the dominant short-range structural units in the glasses containing 25 to 35 mol% of Fe_2_O_3_, the pyrophosphate units are linked by Fe–O units.^19,20^ The Raman spectra of the glass sample 40FeP shows some differences, it has peak intensity at ∼1002 cm^−1^ instead at 1169 cm^−1^, the crystalline sample 50FeP also shows maximum intensity at ∼1002 cm^−1^. The peak at 1002 cm^−1^ is due to P–O^−^ stretching vibrations of non-bridging oxygens in *Q*^0^ units in orthophosphate groups. Finally, the shoulder at 1293 cm^−1^ is due to the bond vibrations of metaphosphate (PO_3_)^−^ units.^19,20^ The analysis of the area of the deconvoluted peaks of Raman spectra provides more information about the short-range structural transformation occurring in the iron phosphate glasses as a function of Fe_2_O_3_ concentration. The variation of area of the 11 peaks in the glass samples is shown in Fig. 5. It is found that the maximum area is of the peak at ∼1087 cm^−1^ and the area of this peak decreases significantly from 48 to 24% with an increase in Fe_2_O_3_ concentration from 25 to 40 mol%. This peak is due to symmetric stretching P–O vibrations in *Q*^1^-FeO_6_ units. Similarly, the areas of the peak at 472 cm^−1^ is due to bending vibrations of P–O bonds in *Q*^1^-FeO_6_ units, the area under this peak also decreases steadily. On the other hand, the area under the peaks at 1185 cm^−1^ (symmetric stretching P–O vibrations in *Q*^1^-FeO_5_ units), 1234 cm^−1^ (symmetric stretching P–O stretching vibrations in *Q*^1^-FeO_4_ units) and at 1293 cm^−1^ (asymmetric stretching P–O vibrations in *Q*^1^-FeO_4_) increases steadily (Fig. 5). These results reveal that the average Fe–O co-ordination number decreases with an increase in Fe_2_O_3_ content, a decrease in octahedral co-ordination of Fe with O indicates that the oxidation state of Fe must be decreasing simultaneously from +3 to +2, and this is indeed confirmed from the neutron diffraction and Mössbauer studies discussed below.

Finally, on comparing the Raman spectra of glasses (samples 25FeP, 30FeP, 35FeP and 40FeP) with that of crystalline sample (50FeP), it is found that broad Raman bands in glasses occur at almost same positions as the sharp peaks in the crystalline sample, and hence it is concluded that the short-range structures of iron phosphate glass and crystalline samples are very similar, further the relative concentration of different short-range structural units *i.e. meta*, pyro and *ortho* units varies with an increase in Fe_2_O_3_ concentration.^19,20^

### 3.5 Short-range structure by neutron diffraction PDF and RMC analysis

The neutron scattering weight factors of all the six atomic pairs of glass samples are given in the Table 3. The neutron scattering weight factors of Fe–Fe, Fe–P and P–P atomic pairs are small while that of Fe–O, P–O and O–O atomic pair correlations have significantly higher values; therefore, the coordination number were calculated for the latter three atomic pair correlations.

**Table 3:** Neutron diffraction weighting factors, *w*_*ij*_ (%) of the interatomic correlations in iron phosphate glasses

Atomic pair	Weight factors, *w*_*ij*_ (%)			
	25FeP	30FeP	35FeP	40FeP
Fe–Fe	1.506	2.193	2.987	3.938
Fe–P	4.907	5.547	6.018	6.430
Fe–O	16.628	19.684	22.573	25.383
P–P	3.995	3.507	3.031	2.624
P–O	27.08	24.895	22.742	20.722
O–O	45.882	44.171	42.646	40.901

**Fig. 6:**
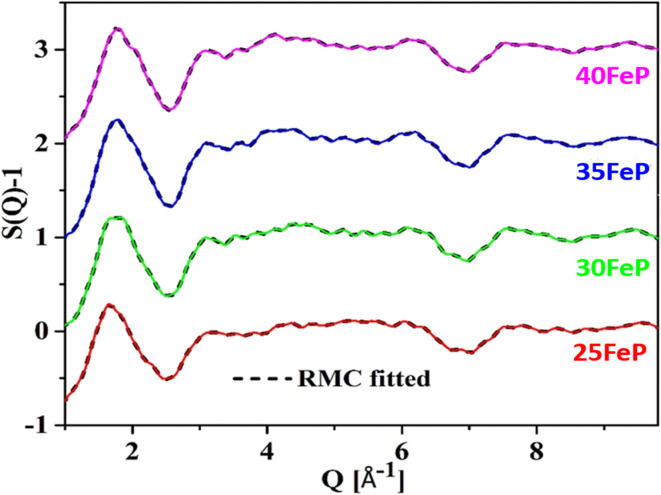
Experimental and RMC calculated neutron structure factors (*S*(*Q*) *−* 1) of 4 iron phosphate glasses. Curves are displaced by 1 unit successively for clarity.

**Fig. 7:**
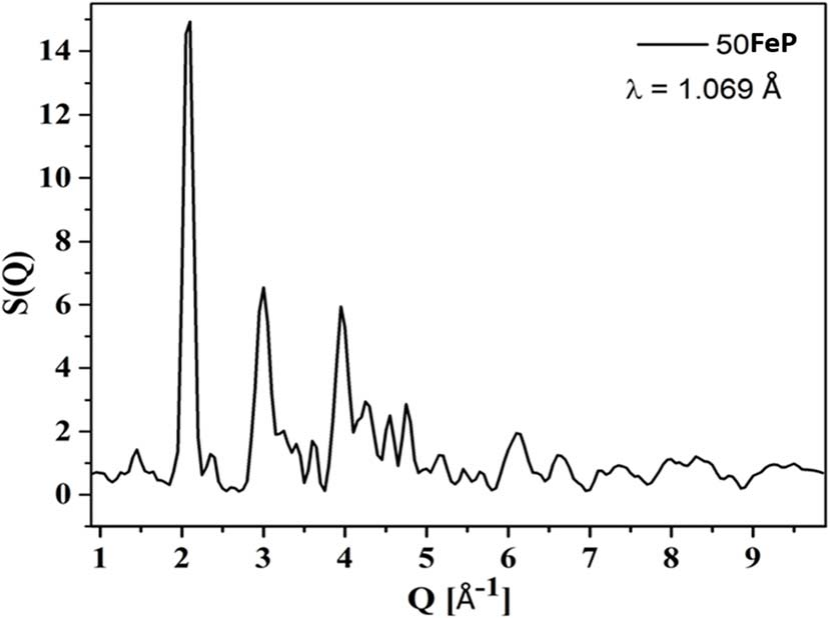
Neutron structure factor, *S*(*Q*) of crystalline iron phosphate sample.

**Fig. 8:**
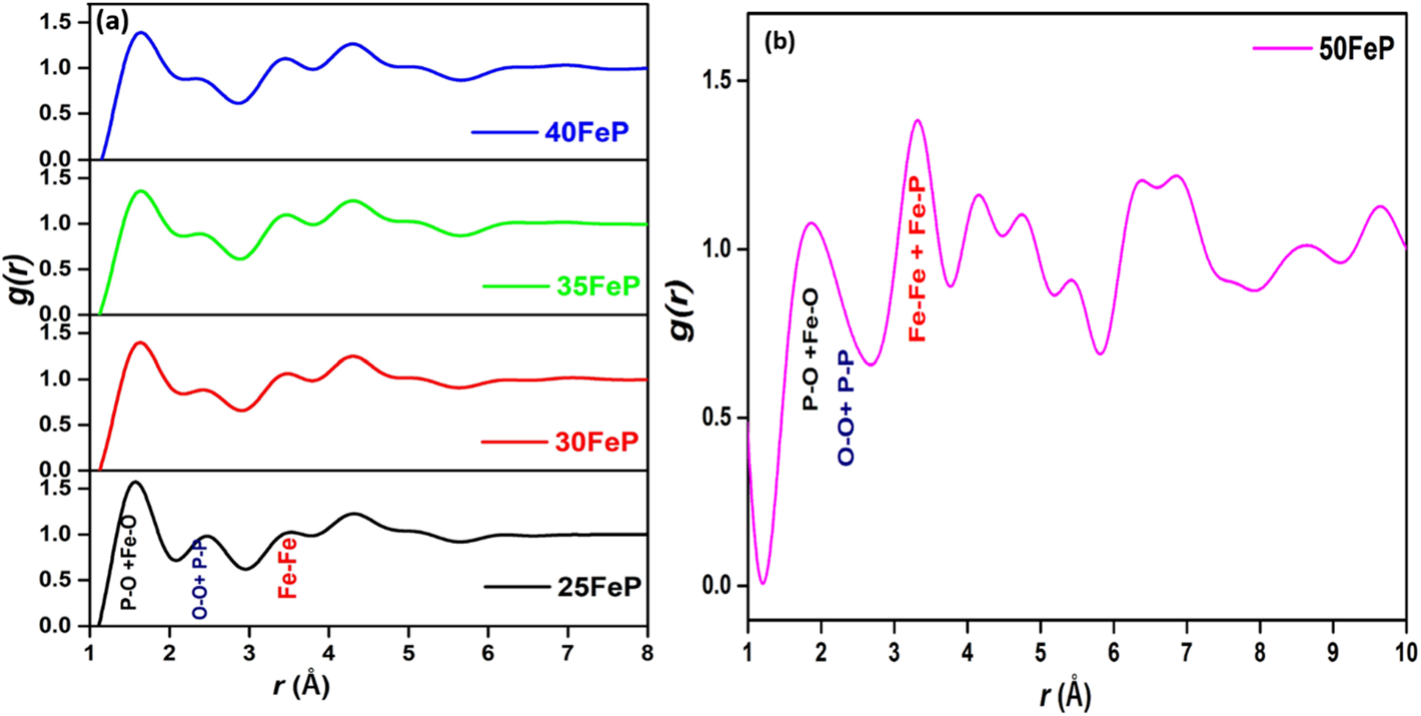
Pair distribution function, *g*(*r*) of iron phosphate glasses (a) 25FeP to 40FeP and (b) crystalline sample (50FeP) calculated by Fourier transformation of *S*(*Q*) with Lorch modification function.

Fig. 6 shows the experimental neutron diffraction (*S*(*Q*) − 1) factors of 4 glass samples together with those calculated by the RMC technique. The experimental structure factors, (*S*(*Q*) − 1) of all the iron phosphate glass samples match well with the calculated structure factors. The neutron diffraction structure factor of the crystalline sample (50FeP) is shown in Fig. 7. The structure factors were used to calculate the pair distribution function, *g*(*r*) by Fourier transformation (eqn (3)) and these are displayed for all samples on Fig. 8a and b.

The first peak in *g*(*r*) of the sample 25FeP is centered at 1.58 Å, this peak shifts to higher values of 1.62–1.65 Å in the samples 30FeP, 35FeP and 40FeP and it is centered at a significantly higher position of 1.84 Å in the crystalline sample 50FeP. This peak is due to superposition of P–O and Fe–O atomic pair correlations, and shifting of its position to longer distances with an increase in Fe_2_O_3_ concentration is due to a rise in the relative concentration of Fe–O atomic pairs in the glass network and also due to enhancement in the relative concentration of Fe^2+^ at the expense of Fe^3+^ (as found by Mössbauer studies discussed later). It is known that Fe^2+^–O bond lengths are longer than those of Fe^3+^–O linkages.^21,28^ The second peak in *g*(*r*) at ∼2.5 Å is due to O–O and P–P correlations and its intensity decreases with a decrease in the concentration of P_2_O_5_ in the samples. The third peak at ∼3.5 Å is due to Fe–Fe atomic pairs and its intensity grows significantly with an increase in Fe_2_O_3_ concentration. The pair distribution function of the sample 50FeP shows oscillations at distances, *r* > 6 Å due to long-range atomic order that exists in the crystalline sample (Fig. 8b). In the case of the crystalline sample 50FeP, the structure factor shows the sharp and intense peak at ∼2 Å^−1^ (Fig. 7), the First Sharp Diffraction Peak is due to medium range order of Fe–Fe and Fe–P atomic pairs. The higher medium range ordering is manifested in *g*(*r*) of the sample 50FeP (Fig. 8b) in which the intensity of the first peak at 1.84 Å is significantly less than that of the second peak at 3.4 Å. It is therefore concluded that Fe–Fe and Fe–P atomic pairs are dominant scattering species in the crystalline sample, whereas in case of glasses, the short range structural features of P–O and Fe–O atomic pairs are the dominant scattering species (Fig. 8a).

**Fig. 9:**
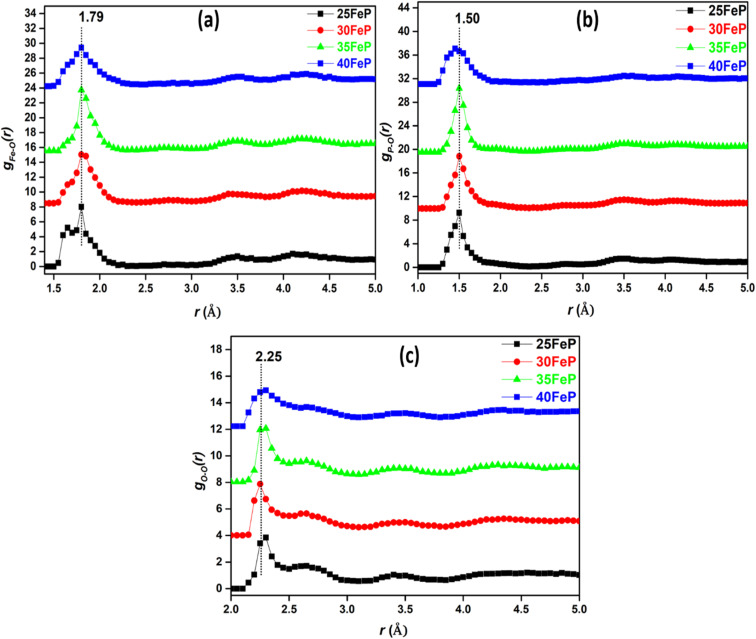
Partial atomic pair correlations of (a) Fe–O (*g*_Fe–O_(*r*)), (b) P–O (*g*_P–O_(*r*)) and (c) O–O (*g*_O–O_(*r*)), in glass samples (curves are shifted successively along the *y* axis by 2.5 units for clarity).

**Fig. 10:**
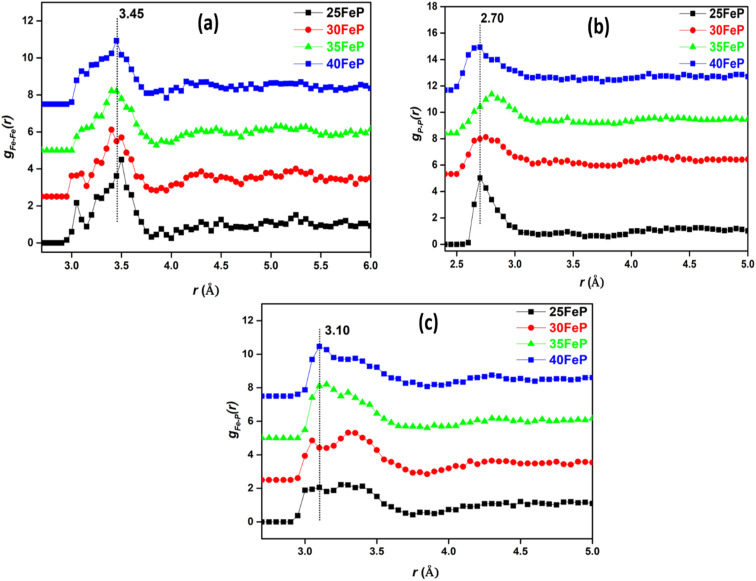
Partial atomic pair correlations of (a) Fe–Fe (*g*_Fe–Fe_(*r*)), (b) P–P (*g*_P–P_(*r*)) and (c) Fe–P (*g*_Fe–P_(*r*)) in glass samples (curves are shifted successively along the *y*-axis by 2.5 units for clarity).

The P–O and Fe–O pair correlations strongly overlap in *g*(*r*) distributions, however the neutron data modelling by RMC technique provided the partial atomic pair distribution functions, *g*_*ij*_(*r*) and the coordination numbers, *N*_*ij*_. The partial pair correlation functions of all the six atomic pairs *i.e. g*_Fe–O_(*r*),*g*_Fe–P_(*r*), *g*_Fe–O_(*r*), *g*_P–P_(*r*),*g*_P–O_(*r*) and *g*_O–O_(*r*) are displayed in Fig. 9a–c and 10a–c.

The *g*_Fe–O_(*r*) has a peak at 1.79 ± 0.05 Å in 25FeP glass sample (Fig. 9a). On increasing the Fe_2_O_3_ concentration from 25 to 40 mol%, the peak position in Fe–O pair correlation remains constant at 1.79 Å (Fig. 9a and Table 4). It is reported that the peaks in the range of 1.80–1.87 Å are due to Fe^3+^–O bonds,^21,28^ and further it is observed that the *g*_Fe–O_ is asymmetrical towards its tail end which indicates that there is significant peak due to Fe^2+^–O bonds, that are reported to show peaks at a longer distance of 2.12 Å in the iron phosphate glass network.^21,28^ Zhang *et al.* found from titration analysis that Fe^2+^/Fe^3+^ concentration ratio in glasses increases with melting time and temperature and with an increase in Fe_2_O_3_ concentration,^19^ these investigators used alumina instead of a more durable platinum crucible for glass melting, the alumina crucibles can introduce Al^3+^ impurities in the final glasses and Al^3+^ can reduce some of Fe^3+^ into Fe^2+^ and produce erroneous results on iron phosphate glass structure and properties. The reported results of increase in Fe^2+^ concentration by Zhang *et al.* in iron phosphate glasses with increase in melting time and temperature can also be due to steady increase in leaching of alumina crucible by iron phosphate melt that will introduce large concentration of Al^3+^ impurities and Al–O correlations in the phosphate network that have bond lengths very close to that of Fe–O linkages.^19^

**Table 4:** Nearest neighbour atomic distances for atomic pairs in iron phosphate glasses. The maximum uncertainty is ±0.05 Å for interatomic distances

Sample code	Interatomic distances, *g*_ij_(*r*) [Å]					
	Fe–Fe	Fe–P	Fe–O	P–P	P–O	O–O
25FeP	3.50	3.08	1.79	2.70	1.50	2.29, 2.75
30FeP	3.41	3.05	1.79	2.76	1.50	2.25, 2.75
35FeP	3.45	3.13	1.79	2.74	1.50	2.29, 2.75
40FeP	3.45	3.10	1.79	2.70	1.48	2.29, 2.70

Hoppe *et al.* found from high energy X-ray diffraction studies that iron phosphate glasses prepared using FeO as a starting material (in the batch mixture) contained mostly Fe^2+^ while the glasses prepared from Fe_2_O_3_ contained mostly Fe^3+^ in the network, it is noteworthy that Hoppe *et al.* used alumina crucible for the synthesis of iron phosphate glasses.^21^ In the present study, Pt crucible, melting temperature of 1300 °C and melting time of ∼1 h has been used for glass synthesis, however large concentration of Fe^2+^ has been found in all samples (from Mössbauer studies discussed below) although Fe_2_O_3_ has been used as the starting material. It is therefore concluded that oxidation state of Fe in the final iron phosphate glass network doesn't seem to depend on the oxidation state of Fe in the starting materials.

The *g*_P–O_(*r*) and *g*_O–O_(*r*) distributions are shown in Fig. 9b and (c) respectively. The P–O partial pair correlation distributions are asymmetrical, and the most probable P–O bond length is in the range: 1.48–1.50 ± 0.05 Å in glasses. The O–O pair distribution is asymmetrical and there are at least two O–O distances of 2.27 ± 0.05 Å and 2.6 ± 0.05 Å in the phosphate network (Table 4). The Fe–Fe, Fe–P and P–P partial correlation functions are shown in Fig. 10a–c. The Fe–Fe partial pair distribution function, *g*_Fe–Fe_(*r*), shows peak at 3.45 ± 0.05 Å (Fig. 10a). The P–P and Fe–P atomic pair correlation distributions shows the first peaks in the ranges: 2.70–2.90 ± 0.05 Å and 3.10–3.30 ± 0.05 Å, respectively. Within the sample series, the shape of the atomic pair distributions are quite similar to each other.

**Fig. 11:**
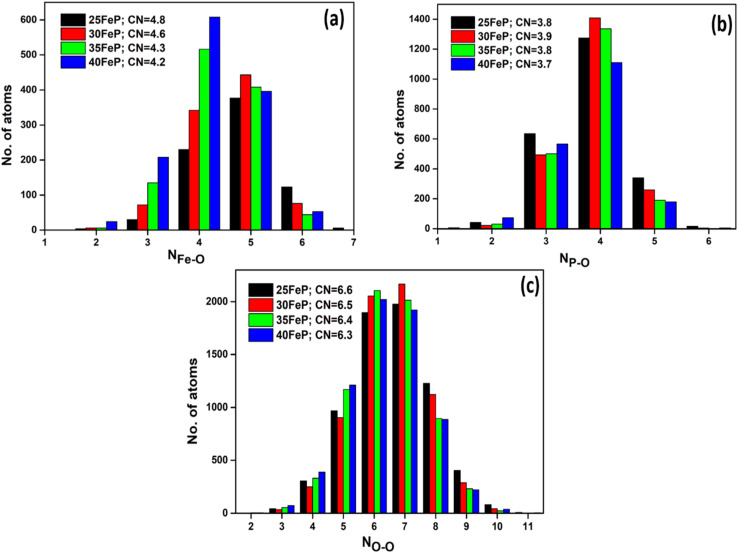
(a) Fe–O (b) P–O and (c) O–O co-ordination distributions in iron phosphate glasses.

The cation-oxygen co-ordination numbers were calculated from the *g*_*ij*_(*r*) plots and the corresponding *r*_min_ and *r*_max_ values that were used to calculate co-ordination number data are given in Table 5. The average co-ordination number of iron with oxygen (*N*_Fe–O_) is 4.8 ± 0.1 in the 25FeP sample, it decreases steadily to a value of 4.2 in the glass sample 40FeP (Fig. 11a). Earlier Stoch *et al.*^28^ found from *ab initio* simulations studies on 40Fe_2_O_3_–60P_2_O_5_ glass that the short-range structure of this glass is very similar to that of crystalline FePO_4_ and basic building blocks are tetrahedral FeO_4_ and PO_4_ units.

**Table 5:** Co-ordination numbers in iron phosphate glasses. The values of *r*_min_ and *r*_max_ used to calculate the coordination numbers in glasses are given in brackets

Sample code	Co-ordination number, *N*_*ij*_ (±0.1)		
	Fe–O	P–O	O–O
25FeP	4.8 (1.30–2.40)	3.8 (1.30–2.35)	6.6 (2.10–3.10)
30FeP	4.6 (1.40–2.35)	3.9 (1.20–2.30)	6.5 (2.00–3.10)
35FeP	4.3 (1.40–2.35)	3.8 (1.30–2.35)	6.4 (2.10–3.10)
40FeP	4.2 (1.40–2.35)	3.7 (1.30–2.35)	6.3 (2.00–3.10)

The P–O coordination (*N*_P–O_) is in the range of 3.8–3.7(±0.1) (Fig. 11b), therefore it is concluded that the P is essentially tetrahedrally co-ordinated with oxygens in the iron phosphate network. Finally, in case of O–O correlations, the average coordination number (Fig. 11c) is found to be in the range of 6.6–6.3 (±0.1). A steady decrease in Fe–O co-ordination number with an increase in Fe_2_O_3_ mol% is due to increasing oxygen deficiency in the glass network which also slightly lowers the P–O and O–O co-ordination numbers.

### 3.6 Fe oxidation states

**Fig. 12:**
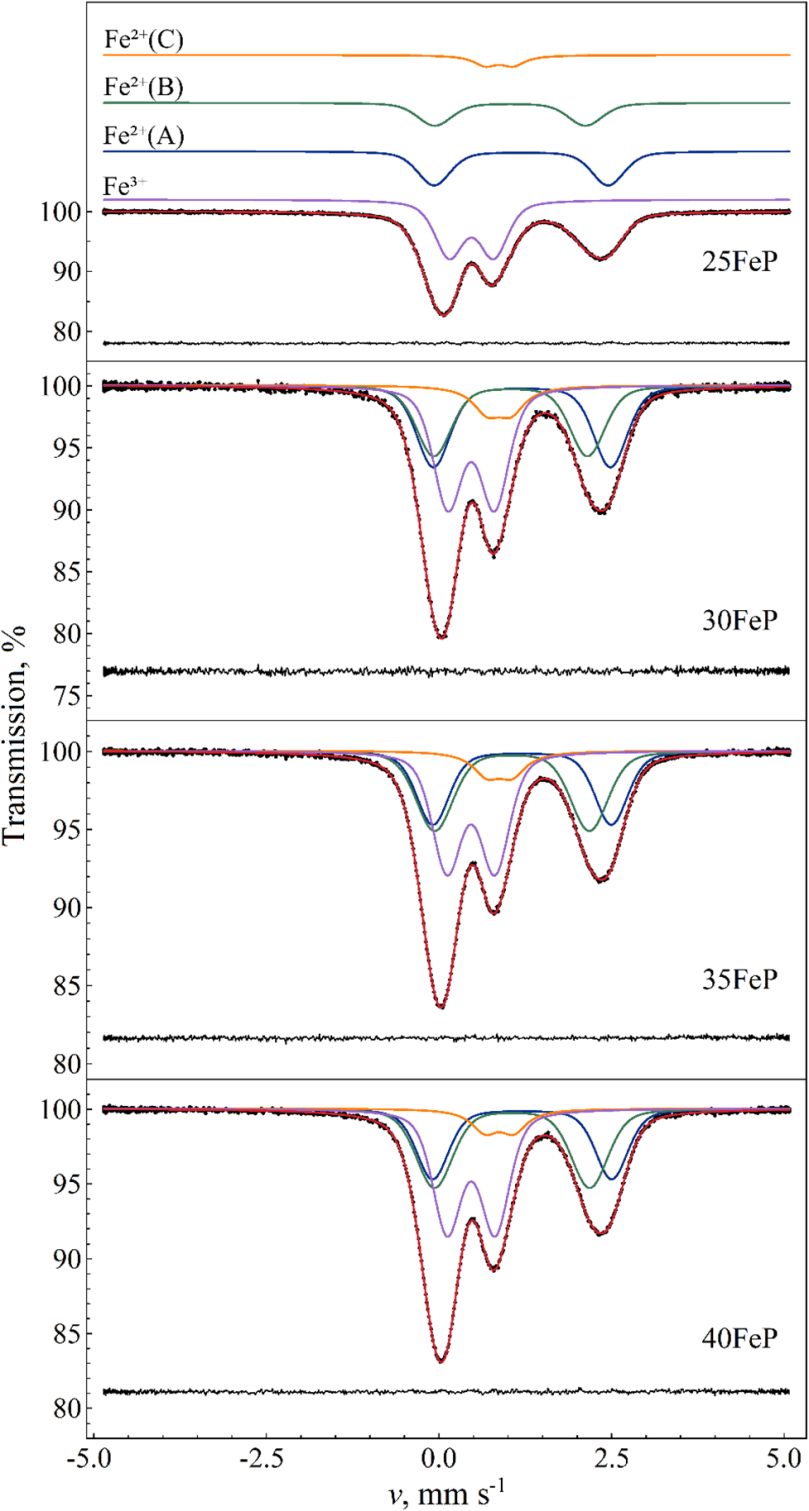
Room temperature ^57^Fe Mössbauer spectra (dots) of amorphous 25FeP, 30FeP, 35FeP and 40FeP samples, along with their fit envelope and decomposition of the resonant absorption peaks into subcomponents (solid line). For the sample 25FeP the subcomponents are shifted upwards for clarity. The fit residual is shown below the spectra. See Table 6 for the associated fit parameter values.

The ^57^Fe Mössbauer spectra of the four glass samples (*i.e.* those with *x* = 25, 30, 35, 40) recorded at room temperature are displayed on Fig. 12. The spectra allowed us to distinguish one Fe^3+^ along with three different main Fe^2+^ iron microenvironments, each contributing to the spectra with a distinct quadrupole doublet component. The spectral shape indicates that the individual absorption peaks belonging to the doublet components are subject to non-Lorentzian broadening. This is due to a distribution in the isomer shift (*δ*) and/or in the quadrupole splitting (*Δ*) Mössbauer parameters, reflecting the presence of a high number of slightly different iron microenvironments associated with the different Fe^2+^ and Fe^3+^ species, which is indeed a typical trait of glasses. The spectra were fitted successfully with Voigt-based fitting^57^ by assuming a Gaussian distribution to be present only in the quadrupole splitting values of the doublets, except in the case of component Fe^2+^(C) (Fig. 12), for which the peaks' profile was better modelled by pure Lorentzian broadening. The Mössbauer parameters obtained from the fits are listed in Table 6.

**Table 6:** Room temperature ^57^Fe Mössbauer parameters of *x*Fe_2_O_3_·(100 − *x*)P_2_O_5_ (*x* = 25, 30, 35 and 40) glass samples obtained by fitting the spectra to four quadrupole doublets with a possible Gaussian distribution in the quadrupole splitting parameters being taken into account *via* Voigt-based fitting.^57^ Numbers between parentheses denote the statistical error (1*σ*) in the last digit(s), whereas (F) refers to fixed parameter values. RA stands for the relative area fraction, *δ* denotes the ^57^Fe isomer shift (wrt. α-Fe at room temperature), 〈*Δ*〉 denotes the mean and *σ*(*Δ*) denotes the standard deviation of the Gaussian distribution assumed to be present in the quadrupole splitting values*,* and *W* is the Lorentzian FWHM width of the individual peaks of the doublets. *χ*_*n*_^2^ denotes the normalized chi-square of the fits

Sample code	25FeP	30FeP	35FeP	40FeP
*x*	25	30	35	40
*χ*_*n*_^2^	1.20	1.07	1.04	1.10

**Quadrupole doublet Fe** ^**2+**^ **(A)**				
RA, %	29.2(2.3)	28.6(3.5)	25.1(2.7)	24.3(2.4)
*δ*, mm s^−1^	1.19(3)	1.20(5)	1.20(1)	1.21(1)
〈*Δ*〉, mm s^−1^	2.52(6)	2.6(1)	2.59(4)	2.60(4)
*σ*(*Δ*), mm s^−1^	0.35(1)	0.36(2)	0.36(1)	0.35(1)
*W*, mm s^−1^	0.256(6)	0.255(9)	0.245(6)	0.249(5)

**Quadrupole doublet Fe** ^**2+**^ **(B)**				
RA, %	20.4(2.3)	25.5(3.5)	30.2(2.8)	30.1(2.4)
*δ*, mm s^−1^	1.03(5)	1.04(7)	1.06(2)	1.06(2)
〈*Δ*〉, mm s^−1^	2.18(8)	2.2(1)	2.24(3)	2.25(3)
*σ*(*Δ*), mm s^−1^	0.37(2)	0.38(2)	0.42(1)	0.41(1)
*W*, mm s^−1^	0.256(6)	0.255(9)	0.245(6)	0.249(5)

**Quadrupole doublet Fe** ^**2+**^ **(C)**				
RA, %	7.4(3)	8.7(5)	7.9(4)	7.5(3)
*δ*, mm s^−1^	0.872(6)	0.87(1)	0.877(9)	0.875(7)
〈*Δ*〉, mm s^−1^	0.40(1)	0.34(2)	0.36(2)	0.41(2)
*σ*(*Δ*), mm s^−1^	0(F)	0(F)	0(F)	0(F)
*W*, mm s^−1^	0.41(1)	0.49(3)	0.49(2)	0.47(2)

**Quadrupole doublet Fe** ^**3+**^				
RA, %	43.0(2)	37.1(4)	36.9(3)	38.2(3)
*δ*, mm s^−1^	0.470(2)	0.467(4)	0.464(3)	0.466(3)
〈*Δ*〉, mm s^−1^	0.640(4)	0.673(7)	0.693(8)	0.693(6)
*σ*(*Δ*), mm s^−1^	0.286(5)	0.298(8)	0.310(6)	0.298(5)
*W*, mm s^−1^	0.256(6)	0.255(9)	0.245(6)	0.249(5)

Around 40% of the spectral area is associated with iron in the high-spin (HS) Fe^3+^ state in each case. With increasing Fe_2_O_3_ mol% the relative area fraction of the Fe^3+^ component drops by *ca.* 6% (from ∼43% to ∼37%) as the Fe_2_O_3_ concentration increases from 25% to 30% and above, which can refer to a simultaneously increased level of oxygen deficiency in the glass structure. The Fe^3+^ isomer shift and mean quadrupole splitting Mössbauer parameters, *δ* ≈ 0.47 mm s ^−1^ and 〈*Δ*〉 ≈ 0.64–0.69 mm s ^−1^ (Table 6), are consistent with octahedral oxygen coordination of high-spin Fe^3+^.

**Fig. 13:**
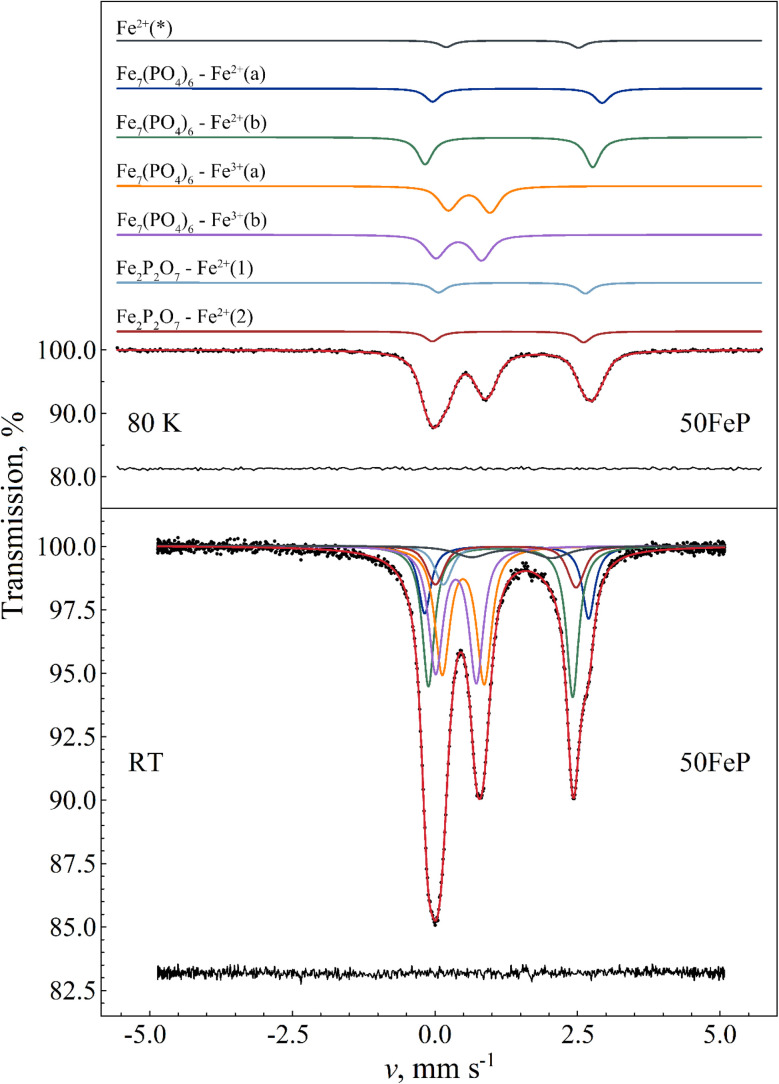
^57^Fe Mössbauer spectrum (dots) of 50Fe_2_O_3_·50P_2_O_5_ (50FeP) crystalline sample measured at 80 K (top) and at room temperature (bottom), along with the indication of the fit envelope and the subcomponents (solid line). For the measurement performed at 80 K, the fitted subcomponents are shifted upwards for clarity. Below the spectra the fit residual is displayed. See Table 7 for the associated fit parameter values.

For the assignment of the obtained Fe^3+^ and Fe^2+^ doublet components it is informative to compare the corresponding Mössbauer parameters with those obtained for Fe_7_(PO_4_)_6_ in the crystalline sample 50FeP (Table 7). The ^57^Fe Mössbauer spectra of the latter sample measured at room temperature and at *T* = 80 K are shown in Fig. 13. Based on the associated X-ray diffractometry results (see Fig. 1) we assumed paramagnetic components of Fe_7_(PO_4_)_6_ and Fe_2_P_2_O_7_ to contribute to the spectra. The spectral decomposition was carried out accordingly, by taking into account the results of previous studies concerning Fe_7_(PO_4_)_6_ (ref. 58) and Fe_2_P_2_O_7_,^59^ and applying suitable constraints among others regarding the relative area fractions of the individual subcomponents. To fit these spectra properly in the thin absorber approximation limit, a minor asymmetry in the peak areas associated with the individual doublets had to be assumed (Table 7), which may be the consequence of thickness effects. The results indicate that in the sample 50FeP *ca.* 79% of iron is incorporated in the lattice of Fe_7_(PO_4_)_6_, and *ca.* 16% in Fe_2_P_2_O_7_ (Table 7), while a further *ca.* 5% contributes to a minor Fe^2+^ species (denoted with Fe^2+^(*) on Fig. 13) that remains unassigned. On the basis of the relatively low quadrupole splitting of the latter component, it may originate from Fe^2+^ species situated in a non-regular, distorted oxygen coordination environment. In the lattice of Fe_7_(PO_4_)_6_, Fe^2+^ occur with 6-fold (octahedral) and 5-fold (pyramidal) oxygen coordination^60^ with a relative occurrence of 2 : 1 in favor of the 5-fold coordination. On the basis of its higher relative area fraction and lower isomer shift value, among the Fe^2+^ components associated with Fe_7_(PO_4_)_6_ (*i.e.* Fe^2+^(a) and Fe^2+^(b) in Table 7) in the spectra of 50FeP it is the Fe^2+^(b) component that can be associated with the 5-fold coordinated Fe^2+^ species. In addition to the Fe^2+^ sites, the structure of Fe_7_(PO_4_)_6_ also includes two distinct Fe^3+^ sites.^60^ In both of these Fe^3+^ sites, the iron atoms exhibit a distorted octahedral oxygen coordination. The Mössbauer parameters we obtained for these sites are given under the names Fe_7_(PO_4_)_6_–Fe^3+^(a) and Fe^3+^(b) in Table 7.

**Table 7:** ^57^Fe Mössbauer parameters of the crystalline 50FeP sample measured at room temperature (RT) as well as at *T* ≈ 80 K. The quadrupole doublets fitted to these spectra were asymmetric, *A*_+_/*A*_−_ denoting the ratio of the areas of the higher- and lower-velocity absorption peaks of the doublets. (For a given spectrum the same ratio was applied to all the fitted doublets.) Relative area fractions of Fe_7_(PO_4_)_6_ components were constrained (at both temperatures) in accordance with the results of Millet *et al.*^58^ On the basis of the work by Ericsson *et al.*,^59^ the area fractions of Fe_2_P_2_O_7_ components were assumed to be equal, and an isomer shift difference of 0.07 mm s^−1^ was assumed between the two doublets. Furthermore, in the room-temperature spectrum the quadrupole splitting of the two Fe_2_P_2_O_7_ doublets were constrained in such a way that ensured that the higher-velocity absorption peaks of the doublets coincide with each other.^59^ See Table 6 for notations

Sample code	50FeP	
*T*, K	RT	80
*χ*_*n*_^2^	1.15	1.08
*A*_+_/*A*_−_	1.08(1)	1.10(1)

**Fe**_**7**_ **(PO**_**4**_ **)**_**6**_ **–Fe** ^**2+**^ **(a)**		
RA, %	11.1(1)	11.0(1)
*δ*, mm s^−1^	1.258(3)	1.45(4)
〈*Δ*〉, mm s^−1^	2.874(6)	2.97(8)
*σ*(*Δ*), mm s^−1^	0(F)	0(F)
*W*, mm s^−1^	0.261(4)	0.309(6)

**Fe**_**7**_ **(PO**_**4**_ **)**_**6**_ **–Fe** ^**2+**^ **(b)**		
RA, %	23.0(2)	22.8(2)
*δ*, mm s^−1^	1.151(3)	1.298(3)
〈*Δ*〉, mm s^−1^	2.529(4)	2.939(5)
*σ*(*Δ*), mm s^−1^	0(F)	0(F)
*W*, mm s^−1^	0.261(4)	0.309(6)

**Fe**_**7**_ **(PO**_**4**_ **)**_**6**_ **–Fe** ^**3+**^ **(a)**		
RA, %	23.0(2)	22.8(2)
*δ*, mm s^−1^	0.50(1)	0.60(1)
〈*Δ*〉, mm s^−1^	0.74(1)	0.73(1)
*σ*(*Δ*), mm s^−1^	0.10(1)	0.14(3)
*W*, mm s^−1^	0.261(4)	0.309(6)

**Fe**_**7**_ **(PO**_**4**_ **)**_**6**_ **–Fe** ^**3+**^ **(b)**		
RA, %	22.2(2)	22.0(2)
*δ*, mm s^−1^	0.37(1)	0.42(3)
〈*Δ*〉, mm s^−1^	0.71(1)	0.80(7)
*σ*(*Δ*), mm s^−1^	0.09(1)	0.14(3)
*W*, mm s^−1^	0.261(4)	0.309(6)

**Fe**_**2**_ **P**_**2**_ **O**_**7**_ **–Fe** ^**2+**^ **(1)**		
RA, %	7.9(3)	8.2(2)
*δ*, mm s^−1^	1.31(2)	1.35(5)
〈*Δ*〉, mm s^−1^	2.33(4)	2.6(1)
*σ*(*Δ*), mm s^−1^	0.15(2)	0(F)
*W*, mm s^−1^	0.261(4)	0.309(6)

**Fe**_**2**_ **P**_**2**_ **O**_**7**_ **–Fe** ^**2+**^ **(2)**		
RA, %	7.9(3)	8.2(2)
*δ*, mm s^−1^	1.24(2)	1.28(5)
〈*Δ*〉, mm s^−1^	2.47(4)	2.7(2)
*σ*(*Δ*), mm s^−1^	0.15(2)	0(F)
*W*, mm s^−1^	0.261(4)	0.309(6)

**Fe** ^**2+**^ (*)		
RA, %	5(1)	5(F)
*δ*, mm s^−1^	1.35(5)	1.36(3)
〈*Δ*〉, mm s^−1^	1.4(1)	2.32(5)
*σ*(*Δ*), mm s^−1^	0(F)	0(F)
*W*, mm s^−1^	0.7(1)	0.28(5)

The Fe^3+^ isomer shift and mean quadrupole splitting Mössbauer parameters obtained for the glass samples are *δ* ≈ 0.47 mm s^−1^ and 〈*Δ*〉 ≈ 0.64–0.69 mm s^−1^ (Table 6). These values are consistent with octahedral oxygen coordination of high-spin Fe^3+^. They are also similar to the parameters of the Fe^3+^(a) and Fe^3+^(b) species of Fe_7_(PO_4_)_6_ (Table 7), with a closer match to the Fe^3+^(a) component. This corroborates that Fe^3+^ ions have distorted octahedral oxygen coordination in these glasses. The distortion of the oxygen octahedra appears to have a wide range of variability in the glass, as reflected by the considerable standard deviation of the distribution of quadrupole splitting values (Table 6). Note that some of the Gaussian broadening, which we modeled as a distribution in the quadrupole splitting values, may also be the result of a distribution in the isomer shift values.

Similarity between the *δ* and 〈*Δ*〉 values of Fe^2+^(b) in Fe_7_(PO_4_)_6_ and Fe^2+^(A) in the glass samples (Table 6) then suggests that Fe^2+^(A) may be associated mainly with 5-fold coordinated high-spin Fe^2+^ species in the glass. At the same time, contribution of Fe^2+^ species with 6-fold (octahedral) oxygen coordination to the Fe^2+^(A) component cannot be fully excluded, as such a species may also display similar *δ* and 〈*Δ*〉 Mössbauer parameter values; one such example is the Fe^2+^(2) species in Fe_2_P_2_O_7_ (Table 7) where iron has octahedral oxygen coordination.^59^

Considering the Fe–O coordination distribution function displayed in Fig. 11a, component Fe^2+^(B) in Table 6 needs then to be attributed mainly to 4-fold (*e.g.* tetrahedral) coordinated Fe^2+^ species in the glass. The latter attribution is further corroborated by the increasing tendency in the relative area fraction of Fe^2+^(B) with increasing Fe_2_O_3_ mol% (Table 6), which is in qualitative agreement with a similar tendency reflected by the Fe–O coordination distribution function (Fig. 11a).

The isomer shift (*δ* ≈ 0.87 mm s^−1^) and quadrupole splitting (*Δ* ≈ 0.4 mm s^−1^) of the Fe^2+^(C) component found in the spectra of the amorphous samples (Fig. 12) are considerably lower (Table 6) than respective values of Fe^2+^(A) and Fe^2+^(B), and may be attributed to *e.g.*, 4-fold coordinated Fe^2+^ with square-planar oxygen coordination environment. This assignment is also supported by similar isomer shift and quadrupole splitting values observed for square-planar coordinated high-spin Fe^2+^ in the mineral gillespite (*δ* ≈ 0.75 mm s^−1^ wrt. α-Fe, *Δ* ≈ 0.51 mm s^−1^).^61^

**Fig. 14:**
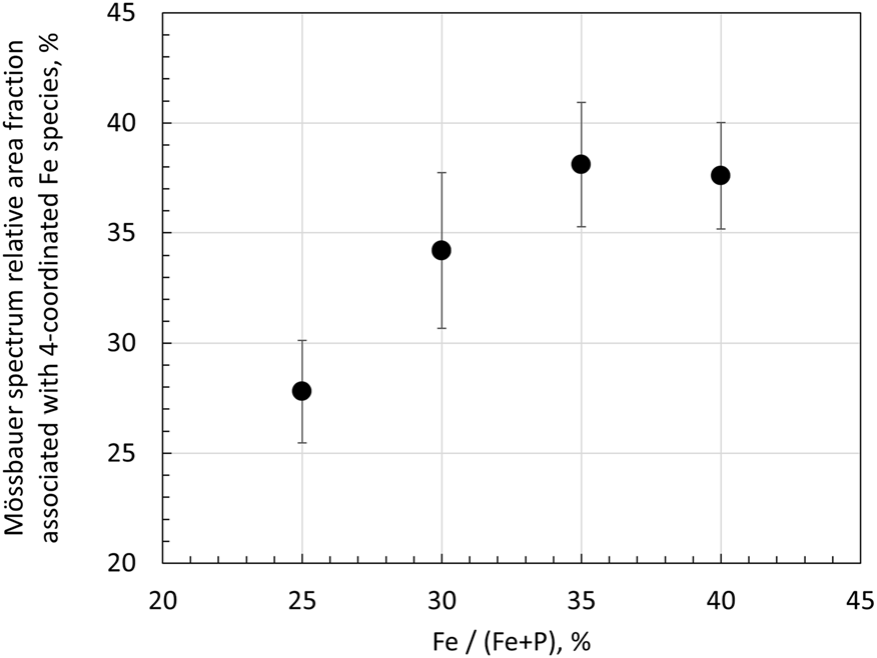
Mössbauer-spectrum relative area fractions associated with 4-coordinated Fe species calculated based on the spectral decomposition of the room-temperature Mössbauer spectra of the 25FeP, 30FeP, 35FeP and 40FeP glass samples (Fig. 12). Among the identified components (Table 6), Fe^2+^ (B) and Fe^2+^ (C) were evaluated to be 4-coordinated species; the figure displays the sum of the relative area fractions of these components as a function of the Fe atomic concentration wrt. the total number of Fe and P atoms in the structure. See Table 6 for the associated Mössbauer parameters. Note that the depicted values were not corrected for possible differences in the recoilless fractions of Fe^2+^ and Fe^3+^ ions.

Fig. 14 displays the sum of the relative area fractions of the Fe^2+^(B) and Fe^2+^(C) doublet components that were attributed to Fe^2+^ species with 4-fold oxygen coordination. Clearly, from 25FeP to 35FeP the relative weight of 4-fold coordinated Fe^2+^ environments increases. On the basis of Table 6, this increase occurs at the expense of both the 5- and 6-coordinated iron species. In line with the model of Hoppe,^62^ the gradual reduction of the coordination number of iron indicates that in the studied samples the concentration of iron already exceeded the limit where the available terminal oxygen ions of PO_4_ tetrahedra could ensure maximum (5–6) coordination number for all the iron ions. Based on the outstanding chemical durability of the 40FeP composition (see Table 9), the development of the glass structure leading to 40FeP must play a decisive role in shaping the advantageous material properties associated with this composition. Based on Fig. 14, from 35FeP to 40FeP the iron coordination numbers seem to be stationary. However, as calculated from atomic pair distribution functions, the concentration of iron atoms with 4-fold oxygen coordination increases further even in this range (Fig. 11a).

**Fig. 15:**
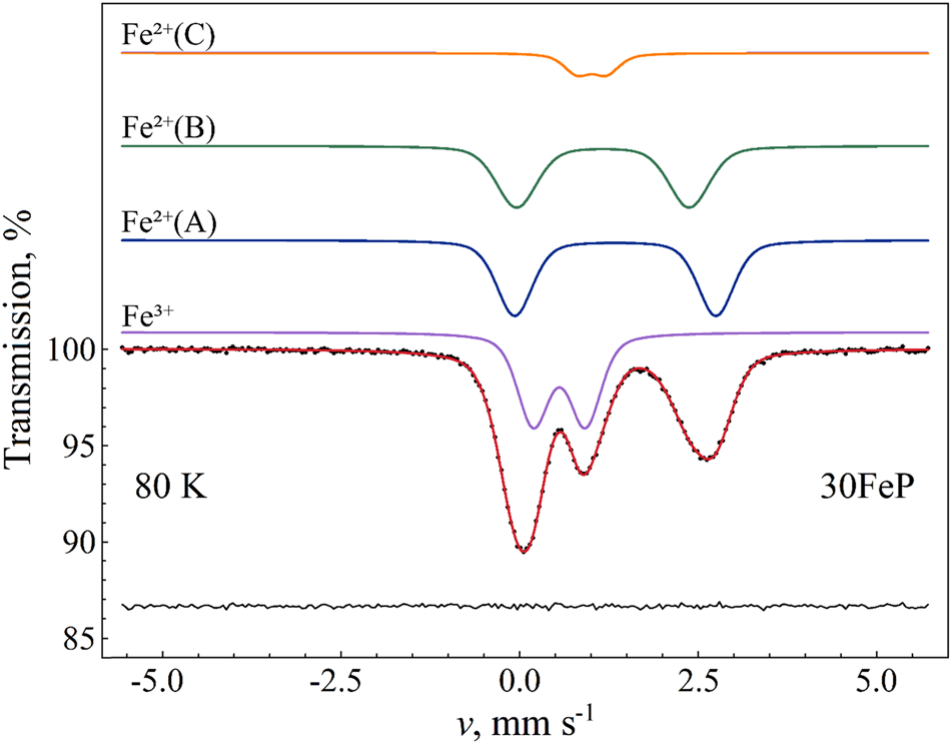
^57^Fe Mössbauer spectrum (dots) of 30Fe_2_O_3_-70P_2_O_5_ (30FeP) glass measured at 80 K, along with the indication of the fit envelope and the subcomponents (solid line). The subcomponents are shifted upwards for clarity. Below the spectrum the fit residual is displayed. See Table 8 for the associated fit parameter values.

Due to possible differences in the temperature dependence of the *f*(Fe^2+^) and *f*(Fe^3+^) recoilless fractions associated with Fe^2+^ and Fe^3+^ species respectively, the Fe^2+^/Fe^3+^ ratio in the glass may not be faithfully reflected by the corresponding relative spectral area ratios characteristic to the room temperature Mössbauer spectra of the glass samples (Fig. 12 and Table 6). In order to find a more reliable value of the Fe^2+^/Fe^3+^ ratio, the sample 30FeP was also measured at *T* = 80 K. The obtained spectrum and the associated fit parameters are shown in Fig. 15 and Table 8. On the basis of the isomer shift and mean quadrupole splitting (〈*Δ*〉) parameters (Table 8), in the latter spectrum (Fig. 15) one can clearly identify the components associated with the iron species Fe^2+^(A), Fe^2+^(B), Fe^2+^(C) and Fe^3+^. In contrast with the spectra of glasses recorded at room temperature (Fig. 12), which reflect an Fe^2+^/Fe^3+^ spectral area ratio of approximately 1.3–1.7 (Table 6), the relative area fractions of the Fe^2+^ and Fe^3+^ components derived on the basis of the spectrum in Fig. 15 indicate that the Fe^2+^/Fe^3+^ ratio is closer to 2 in these glasses, and hence Fe^2+^ species are clearly in majority concentration in the glass samples (Table 8). Concerning the sample 30FeP in particular, the Fe^2+^/(Fe^2+^+Fe^3+^) ratio changes from 62.9(4)% (at room temperature) to 66.0(4)% (at 80 K), *i.e.* roughly by a factor of *q* ≈ 1.05. However, at 80 K the recoilless fraction of Fe^2+^ may still be smaller than that of Fe^3+^. Indeed, by assuming that at room temperature the *f*(Fe^3+^)/*f*(Fe^2+^) ratio of the recoilless fractions is *ca.* 1.3 as given, *e.g.*, in ^63^, the associated factor turns out to be *q* ≈ 1.09, meaning that in the sample 30FeP the real Fe^2+^/(Fe^2+^ + Fe^3+^) ratio could reach 68–69%.

**Table 8:** ^57^Fe Mössbauer parameters of amorphous 30FeP sample measured at *T* ≈ 80 K. Internal Lorentzian FWHM line width values (*W*) were constrained to be the same for all the subcomponents. See Table 6 for the corresponding room temperature data and further notations

Sample code	30FeP
*χ*_*n*_^2^	1.43

**Quadrupole doublet Fe** ^**2+**^ **(A)**	
RA, %	31.4(3.9)
*δ*, mm s^−1^	1.34(2)
〈*Δ*〉, mm s^−1^	2.82(5)
*σ*(*Δ*), mm s^−1^	0.38(2)
*W*, mm s^−1^	0.26(1)

**Quadrupole doublet Fe** ^**2+**^ **(B)**	
RA, %	28.2(3.9)
*δ*, mm s^−1^	1.17(4)
〈*Δ*〉, mm s^−1^	2.42(6)
*σ*(*Δ*), mm s^−1^	0.44(3)
*W*, mm s^−1^	0.26(1)

**Quadrupole doublet Fe** ^**2+**^ **(C)**	
RA, %	6.4(3)
*δ*, mm s^−1^	1.01(2)
〈*Δ*〉, mm s^−1^	0.41(3)
*σ*(*Δ*), mm s^−1^	0.23(2)
*W*, mm s^−1^	0.26(1)

**Quadrupole doublet Fe** ^**3+**^	
RA, %	34.0(4)
*δ*, mm s^−1^	0.556(7)
〈*Δ*〉, mm s^−1^	0.73(1)
*σ*(*Δ*), mm s^−1^	0.31(1)
*W*, mm s^−1^	0.26(1)

The theoretical model we applied for the decomposition of the iron phosphate glass Mössbauer spectra was chosen on account of being the simplest model providing reasonable and consistent results along with excellent mathematical fits (Table 6 and Fig. 12). Nevertheless, the model we applied appears to be rather uncommon in related literature, making it pertinent to compare our results with those obtained using other approaches to the decomposition of Mössbauer spectra of iron phosphate glasses. One of the main difficulties in analyzing the spectra of these glasses is that the absorption peaks are non-Lorentzian, making traditional spectrum analysis techniques based on a few Lorentzian doublets inaccurate. Therefore, different solutions have been developed to address this problem and to extract information from the Mössbauer spectra of such glasses.

The most straightforward approach is to fit glass spectra, akin to those in Fig. 12, with one broadened Lorentzian doublet for Fe^2+^ and one for Fe^3+^,^29,64–66^ which will provide approximate average Mössbauer parameter values for the two different iron oxidation states. While not suitable to extract fine details from spectra of iron phosphate glass where Fe^2+^ or Fe^3+^ ions exist with multiple oxygen coordination environments, this method may be justified especially when the signal-to-noise ratio of the spectrum is not high enough to reveal deviations from the Lorentzian line profile. It also has the advantage of being easily reproducible in different laboratories, facilitating the comparison of associated results. However, even in this simple case two different solutions may be found reasonable regarding the Mössbauer parameters: one in which Fe^3+^ has a higher quadrupole splitting (QS), and therefore the left-most absorption peak belongs to Fe^3+^ in the fit model, and another in which Fe^3+^ has a lower QS, and the left-most Lorentzian of the model belongs to Fe^2+^ (similarly to the cases shown in Fig. 12). Usually, the former model provides the better fit, leading to room temperature Fe^3+^ quadrupole splitting values around 0.8–0.9 mm s^−1^,^29,64,65^ in contrast with the lower mean Fe^3+^ quadrupole splitting values of 0.64–0.70 mm s^−1^ we obtained (Table 6). However, when we apply the same model to the spectrum of 40FeP shown in Fig. 12 (which composition is also studied for example in ref. 22, 29, 63 and 66–68), we also find the larger QS solution to provide the better fit with main Mössbauer parameters of *δ* (Fe^3+^) ≈ 0.38 mm s^−1^, *Δ*(Fe^3+^) ≈ 0.89 mm s^−1^, *δ*(Fe^2+^) ≈ 1.23 mm s^−1^, *Δ*(Fe^2+^) ≈ 2.23 mm s^−1^. At the same time, the more accurate multi-component fits, displayed in Fig. 12, already favor the solution with the lower QS values of Fe^3+^ given in Table 6.

Another common method is the formal fit of the Fe^2+^ and Fe^3+^ spectrum parts with multiple (usually 3–4 for each oxidation state) broadened Lorentzian quadrupole doublets, without associating specific iron species with the individual components. The number of doublets is chosen to be high enough to achieve an acceptable fit of the spectra. The results are then reported as weighted averages of the isomer shift (IS) and quadrupole splitting values obtained for the individual oxidation states.^22,63,67–71^ An advantage of this method over the previous one is that due to the better fit of the spectra the obtained averages can reflect minor changes in the spectral shape more sensitively than by using only a single doublet for each oxidation state.

The main limitation of the two methods mentioned above is that they provide only a single average isomer shift and quadrupole splitting value for each oxidation state. This means that they cannot distinguish between different Fe^2+^ (or Fe^3+^) oxygen coordination environments that are simultaneously present in the sample. The average IS and QS values resulting from such analyses of Mössbauer spectra of iron phosphate glasses are typically found to refer mainly to 6-fold (octahedral or distorted octahedral) oxygen coordination for both Fe^2+^ and Fe^3+^ ions.^22,29,63,66,68–71^

A third method applied to decompose Mössbauer spectra of iron phosphate glasses utilizes the VBF (Voigt based fitting,^72^) or xVBF (extended Voigt based fitting^57^) method to fit quadrupole doublets by assuming correlated Gaussian distributions of the QS and IS Mössbauer parameter values of the individual doublets.^28,73–75^ This is the same method we also apply in the present work, with the significant difference that we have assumed the distributions of IS and QS values to be uncorrelated (or that of IS values being negligible). In the presence of IS-QS correlation the VBF and xVBF models can produce a wide variety of doublet peak profiles, where the amplitudes and the widths of the individual (Voigt-profile) peaks contributing to the doublet can differ from each other. By adjusting the parameter of correlation between distributions of IS and QS values of individual doublets of a fit model, iron phosphate glass spectra could be accounted for with high accuracy with only three main VBF or xVBF components and associated three main iron positions, two assumed for Fe^3+^ and one for Fe^2+^.^28,73–75^ By using this method, in different iron phosphate glass samples Fe^3+^ and Fe^2+^ ions were associated with positions having 6-, 5- and 4-fold and 6- and 5-fold oxygen coordination, respectively. As we see it, currently it is unclear whether correlation between distributions of IS and QS values of iron species should indeed be taken into account in the interpretation of iron phosphate glass Mössbauer spectra. Our results indicate (Fig. 12 and Table 6) that excellent fits can be achieved by assuming only four main iron species, even without considering the possibility of IS-QS distribution correlations. In comparison with previous reports, a novel component in our decomposition of the iron phosphate glass Mössbauer spectra (Fig. 12) is the minor doublet Fe^2+^(C) (Table 6) that displays Mössbauer parameters referring to Fe^2+^ in a square-planar oxygen coordination environment. The inclusion of this component into our model played a decisive role in the achievement of high-accuracy spectrum fits with a limited number of spectrum components.

### 3.7 Chemical durability of iron phosphate glasses

The chemical stability of two bulk glasses of composition 30Fe_2_O_3_–70P_2_O_5_ (sample code 30FeP) and 40Fe_2_O_3_–60P_2_O_5_ (sample code 40FeP) were analyzed over a period of 10 days in MQ-water kept at 90 °C and the concentration of dissolved Fe and P was measured after 1, 2, 3, 7 and 10 days and the experimental data are presented in Table 9. In case of glass sample 30FeP, the concentration of P in MQ-water increases from a value of 1.014 ± 0.100 to 15.855 ± 0.253 mg l^−1^ while that of Fe increases from a value of 0.092 ± 0.006 to 0.747 ± 0.049 mg l^−1^ over a time period of 10 days. In case of 40FeP glass, the concentration of P increases from a value of 0.337 ± 0.039 to 1.231 ± 0.195 mg l^−1^ while that of Fe remains nearly constant in the range: 0.078 ± 0.003 to 0.053 ± 0.013 mg l^−1^. It is found that the chemical stability of 40FeP glass is drastically higher than that of 30FeP glass and further the concentration of dissolved P is significantly higher than that of Fe. These findings are in agreement with the earlier reports that 40Fe_2_O_3_–60P_2_O_5_ glass has maximum chemical durability.^22–26^

**Table 9:** Concentration of P and Fe leached from 30FeP and 40FeP glass samples kept in MQ-water at 90 °C over a period of 10 days

Sample code & number of days	P (mg l^−1^)	Fe (mg l^−1^)
30FeP	1.014 ± 0.100	0.092 ± 0.006
1 Day		
40FeP	0.337 ± 0.039	0.078 ± 0.003
1 Day		
30FeP	6.699 ± 0.100	0.343 ± 0.008
2 Days		
40FeP	0.552 ± 0.074	0.086 ± 0.015
2 Days		
30FeP	11.755 ± 0.188	0.506 ± 0.019
3 Days		
40FeP	0.958 ± 0.033	0.069±±0.010
3 Days		
30Fe	12.807 ± 0.358	0.579 ± 0.0139
7 Days		
40FeP	1.163 ± 0.060	0.069 ± 0.006
7 Days		
30FeP	15.855 ± 0.253	0.747 ± 0.049
10 days		
40FeP	1.231 ± 0.195	0.053 ± 0.013
10 Days		

## 4. Conclusions

Iron phosphate glasses with 25 to 40 mol% Fe_2_O_3_ were produced by melt quenching. The sample with a higher concentration of 50 mol% Fe_2_O_3_ has poor glass-forming ability and it produces crystalline sample containing Fe_7_[PO_4_]_6_ and Fe_2_P_2_O_7_ phases on melt-quenching. The glass transition, crystallization and liquidus temperatures were determined by DSC analysis. The ionic packing fraction show a small variation while the glass forming tendency decreases significantly with an increase in Fe_2_O_3_ concentration in the phosphate network. The RMC analysis of neutron diffraction datasets were used to calculate the partial atomic pair distributions and coordination environments in iron phosphate glasses. The most probable P–O and Fe–O bond distances in glasses are 1.50 Å and 1.79 ± 0.05 Å respectively. Mössbauer studies revealed that Fe exists in both 3+ and 2+ oxidation states in the glasses, with a Fe^2+^/Fe^3+^ occurrence ratio of *ca.* 2. Fe^3+^ ions were identified as having distorted octahedral oxygen coordination, while Fe^2+^ ions in the glass were found to have 4-fold and 5-fold oxygen coordination environments. The relative fraction of Fe^2+^ increases significantly with an increase in Fe_2_O_3_ concentration in the starting bath mixture, which also lowers the glass forming ability of the system. Iron coordination numbers from partial atomic pair distribution functions and Mössbauer results indicated that the occurrence of Fe^2+^ with 4-fold oxygen coordination increases with the concentration of Fe_2_O_3_. Neutron diffraction and Raman studies found that the short-range structure of iron phosphate glasses and crystalline phases are similar. Leaching experiments on iron phosphate glasses in purified water kept at 90 °C confirmed that chemical durability is enhanced considerably with an increase in iron concentration in the glass and that P dissolution is higher than that of Fe.

## Data availability

All data reported in the manuscript is available with the authors and will be provided on request to the corresponding author.

## Conflicts of interest

There are no conflicts to declare.
